# Adjunctive EGb 761 treatment in patients with Parkinson’s disease: a case series

**DOI:** 10.3389/fnagi.2026.1767148

**Published:** 2026-07-17

**Authors:** Dmytro Krasnienkov, Iryna Karaban, Olexiy Barsukov, Veronika Korcheva, Nina Karasevych, Nataliia Melnyk, Maryna Khodakovskaya, Tetiana Papurina, Bohdan Falarin, Vitaly Kukharskyy, Kostiantyn Midlovets

**Affiliations:** 1 D. F. Chebotarev Institute of Gerontology of the National Academy of Medical Sciences of Ukraine, Kyiv, Ukraine; 2 M.H. Kholodny Institute of Botany, Kyiv, Ukraine; 3 V.N. Karazin Kharkiv National University, Kharkiv, Ukraine

**Keywords:** EGb 761, *Ginkgo biloba*, mitophagy, MtDNA copy number, oxidative stress, Parkinson’s disease, telomere length

## Abstract

**Background:**

Parkinson’s disease (PD) is the second most common neurodegenerative disease. It is based on a gradual decrease in the number of dopaminergic neurons in the midbrain. Since various pathological processes, including oxidative stress, neuroinflammation, and impaired neuroplasticity, are involved in PD pathogenesis, there is increasing interest in adjunctive therapies that target multiple pathways. This study aims to provide a preliminary assessment of the effects of adjunctive EGb 761 on motor, cognitive, and molecular markers in patients with Parkinson’s disease, to gain insights valuable for the design of future controlled and randomized clinical trials.

**Methods:**

The study included 17 patients with Parkinson’s disease who received EGb 761 for 1 month in the context of baseline antiparkinsonian therapy. Standard clinical and neurophysiological tests were performed: UPDRS, MMSE, MoCA, FAB, BDI-I, Spielberger—Hanin Anxiety Scale and Hoehn and Yahr scale, motor tempo and sensorimotor response; the following molecular genetic markers were determined: relative average telomere length, relative mtDNA copy number; and biochemical markers: LDH, SOD and CAT activity, as well as AGEs, GSH/GSSG and MDA concentrations were determined.

**Results:**

Statistically significant changes in the relative number of mtDNA copies (decrease), GSH/GSSG (decrease) and MDA (increase), as well as a trend towards telomere elongation were observed.

**Conclusion:**

EGb 761 administration in combination with basic symptomatic therapy is associated with improved cognitive and motor functions, and the molecular findings (a decrease in mtDNA copy number alongside a trend toward telomere elongation) are consistent with, though not proof of, the activation of mitophagy. Our preliminary results provide the basis for designing sufficiently powered, randomized and controlled clinical trials with clearly defined primary and secondary endpoints to assess the efficacy and safety of *Ginkgo biloba* extract in the adjunctive treatment of Parkinson’s disease.

## Background

Parkinson’s disease (PD) is a progressive neurodegenerative disease with late onset, characterized primarily by motor symptoms such as bradykinesia, muscle rigidity, postural instability and resting tremor, as well as several non-motor disorders (Wei et al., 2018; Nandi et al., 2019; Parga et al., 2021; Anwar et al., 2024; Abraham et al., 2005). Non-motor symptoms include cognitive and behavioral disorders, autonomic dysfunction: dementia and executive function disorders, impulsivity, depression and sleep disorders (REM sleep behavior disorder), neuropathic pain and nociceptive sensitization, olfactory disturbances, constipation, urinary incontinence (Wei et al., 2018; Chang and Chen, 2020; Cruickshank et al., 2015; Lin Z.-H. et al., 2022; Xiong and Yu, 2018).

Histologically, the pathogenesis of PD is characterized by the increased death of dopaminergic neurons in the compact part of the *substantia nigra pars compacta* (*SNpc*) in the midbrain (Nandi et al., 2019; Lücking et al., 2000; Khan and Ali, 2018; Liu et al., 2023; Nunes and Laranjinha, 2021; SenGupta et al., 2021). This is the central pathological feature of PD, which is recognized as the cause of the clinical signs of PD, and above all, the motor symptoms of this disease (Xiong and Yu, 2018; Dölle et al., 2016; Ortega-Vázquez et al., 2023; Asghar et al., 2022). In PD, there is also a loss of dopaminergic neurons in the *ventral tegmental area*, which causes emotional and cognitive impairment (Leão et al., 2015). Some non-motor symptoms may be based on neurotransmission disorders and loss of other neuronal populations, such as noradrenergic, serotonergic, and cholinergic neurons (Leão et al., 2015; Rocha et al., 2023). Dysfunction is also observed in other subcortical areas of the brain, such as the *olfactory bulb* (*bulbus olfactorius*), the dorsal motor nucleus of the vagus nerve (dorsal motor nucleus of the vagus, *nucleus dorsalis nervi vagi*), basal nucleus of Meinert (*substantia innominata*), *nuclei raphes*, *locus coeruleus* and *hypothalamus* (Lama et al., 2021). Thus, PD is a multisystemic disorder characterized mainly by motor dysfunctions (Asghar et al., 2022; Lama et al., 2021; Yakunin et al., 2014). The result of a long-term illness is disability and premature death (Cruickshank et al., 2015).

The cytological manifestation of PD is the formation of Lewy bodies—cytoplasmic spherical protein eosinophilic hyaline inclusions. Lewy’s bodies contain various neurofilament proteins: *α*-synuclein, ubiquitin, parkin (Liu et al., 2023; Lama et al., 2021; Yakunin et al., 2014; Sanders and Greenamyre, 2013; Li Y. et al., 2022; Chen et al., 2016; Gao et al., 2022; Henrich et al., 2023). Among the proteins deposited in Lewy’s bodies, and the formation of which is associated with them, are complement factors iC3b and C9 (Hällqvist et al., 2024). It should also be noted that *SNpc* dopaminergic neurons possess a lower total mitochondrial mass, with these organelles often being smaller (Chen et al., 2016). The latter is determined by mitochondrial dysfunction, accumulation of mutations in mtDNA, changes in the number of their copies, and the development of oxidative stress.

Age is a significant non-modifiable risk factor for PD. The mechanisms associated with physiological aging are thought to compound or be exacerbated by the etiological factors of the disease itself. Physiological aging is characterized by a gradual decline in the ability to maintain internal homeostasis due to a reduced ability to respond to various endogenous and exogenous stresses (Abdelmegeed et al., 2016). It is also characterized by time-dependent progressive disorders of anatomical and physiological integrity at the cellular, tissue and organ levels (Asghar et al., 2022; Zgutka et al., 2023; Hagman et al., 2021). At the molecular and cellular levels, nine signs of aging should be distinguished: genomic instability, telomere shortening, epigenetic changes, loss of proteostasis, dysregulation of nutrient input into metabolism, mitochondrial dysfunction, stem cell depletion, altered intercellular communication, and cellular senescence (Gonzales et al., 2022).

PD, except for a small proportion (5–10%) of familial forms, is considered an idiopathic disease, i.e., one whose etiological factors remain unidentified (Rocha et al., 2023; Gao et al., 2022; Castelo Rueda et al., 2023; Filograna et al., 2016). However, several pathological processes have been identified that may accelerate neurodegenerative processes, and their management can improve symptoms. These processes are not the primary causes of the disease, but they may contribute to its pathogenesis. Thus, the processes that cause aging are superimposed on several secondary etiological factors of PD, which traditionally include the following molecular-genetic determinants: oxidative stress (oxidative, lipoperoxidative, carbonyl, nitrosative) (Chang and Chen, 2020; Li Y. et al., 2022; D’Amico et al., 2013; Md et al., 2022; Sharma et al., 2020; Giordano et al., 2014), ferroptosis (D’Amico et al., 2013; Miletić-Vukajlović et al., 2020; Wu et al., 2021), dopamine oxidation (Chang and Chen, 2020; Cruickshank et al., 2015; Filograna et al., 2016; Kalra et al., 1992; Nikolova and Mancheva, 2013), accumulation of advanced glycoxidation and lipoxidation end products (AGEs/ALEs) (Zgutka et al., 2023; Sharma et al., 2020; Li et al., 2012; Perrone et al., 2020; D’Cunha et al., 2022), mitochondrial dysfunction (Park et al., 2018; Tresse et al., 2023; Lin et al., 2012), telomere shortening (Li et al., 2019; Ciacka et al., 2020). As well as microenvironmental and tissue-level processes, such as neuroinflammation (Schürks et al., 2014), axonal loss (Burke and O’Malley, 2013), decreased neuroplasticity (Shah et al., 2003; Fehske et al., 2009; Gargouri et al., 2018; Tchantchou et al., 2007), and neurotransmitter metabolism disorders (Lisowska et al., 2026; Yoshitake et al., 2010).

Herbal medicines have been used for several thousand years and can add to the list of biologically active substances used in modern pharmacology (Nowak et al., 2021). One of them is *Ginkgo biloba*, which is claimed to have been used in traditional Chinese medicine for over 600 years (Nowak et al., 2021). Today, it is prescribed for circulatory disorders, balance improvement, tinnitus and vertigo (dizziness), neurosensory dysfunctions and age-related macular degeneration, Alzheimer’s and Parkinson’s diseases, schizophrenia, as well as for improving cognitive abilities, memory and concentration (Nowak et al., 2021; Christen, 2004; Mahadevan and Park, 2008; Suzuki et al., 2004). It is mainly prescribed in the form of a standardized extract from dried *G. biloba* leaves (EGb 761), first developed for pharmaceutical use in Germany in 1965 (Nowak et al., 2021).

The pharmacological effects of EGb 761 are primarily attributed to flavonoids (such as kaempferol and quercetin) and terpenes, including ginkgolides (terpenic lactones) and bilobalide (terpenic trilactone) (Nowak et al., 2021; Mahadevan and Park, 2008). The antioxidant effect is primarily exerted by flavone glycosides, which act as ROS scavengers and chelating ligands of metal ions, as shown by both *in vitro* and *in vivo* experiments (Giordano et al., 2014; Nowak et al., 2021; Christen, 2004; Mahadevan and Park, 2008). Among the ROS that can be absorbed by the substances that make up the extract are hydroxyl radicals (OH˙), peroxyl radicals (ROO˙), superoxide anion radicals (O_2_˙^−^), nitric oxide radicals (NO˙), hydrogen peroxide (H_2_O_2_) and ferric ion species (Mahadevan and Park, 2008).

Despite the long history of *Ginkgo biloba* use and its recognized multi-mechanistic neuroprotective and cognitive-enhancing properties, clinical evidence supporting the use of the standardized extract EGb 761 as an adjunctive therapy for Parkinson’s disease remains limited and often inconsistent. Furthermore, few studies have simultaneously assessed the effect of EGb 761 on key molecular-genetic markers relevant to PD pathology, such as mitochondrial DNA integrity, telomere dynamics, and specific oxidative stress indicators, in patients already undergoing standard anti-Parkinsonian medication. Therefore, to explore the preliminary clinical and biological effects of EGb 761 when added to basic antiparkinsonian therapy, and to generate hypotheses regarding its potential therapeutic mechanisms in a real-world clinical setting, we conducted this single-center case series.

## Methods

### Information about patients

The study included 17 patients with Parkinson’s disease diagnosed according to the British Brain Bank inclusion and exclusion criteria (Hughes et al., 1992), aged 48–76 years, with a disease stage of 2.0–3.0 (Hoehn and Yahr scale). Dopaminergic replacement therapy, which involves the administration of a metabolic precursor of dopamine (L-DOPA), is a standard treatment that is widely used to significantly improve motor symptoms in patients with PD (Dozio et al., 2020). The drug was administered in the setting of basic antiparkinsonian therapy (drugs containing L-DOPA, dopamine receptor agonists, amantadine, MAO-B inhibitors), which was used for 28–30 days before starting EGb 761 treatment and throughout the study period. The dosing regimen of EGb 761^®^ (Dr. Willmar Schwabe GmbH & Co. KG, Karlsruhe, Germany) was a total of 240 mg orally twice daily for 30 days.

### Clinical diagnostics

The standardized international unified Parkinson’s disease rating scale (UPDRS) was used to assess the dynamics of motor disorders. The stages of PD were characterized using the Hoehn and Yahr scale.

### Clinical and neurophysiological tests

The following clinical scales were used for a comprehensive assessment of non-motor functions:

1. Mini-Mental State Examination (MMSE).
2. Montreal Cognitive Assessment (MoCA) (no correction factors were used) (Koshimoto et al., 2023).
3. The Frontal Assessment Battery (FAB) is a test battery for assessing frontal dysfunction.
4. Beck Depression Inventory (BDI-I).
5. Spielberger-Hanin Anxiety Scale (STAI).

### Motor tempo and sensorimotor response

Motor tempo was determined as the time between consecutive presses of the same finger on two keys spaced 20 cm apart on the keyboard. The patient was instructed to press the keys at the fastest possible tempo. The duration of each test was 20 s. Testing was conducted for each hand separately, and then the data were averaged.

The latency period of a simple sensorimotor reaction was defined as the interval between the moment of presentation of a visual signal on a computer monitor and the moment the patient pressed a key in response to the signal. Geometric shapes (circle, square, oval) of red and white colors were used as signals, 20 signals were presented with an interval of 2–4 s. Testing was conducted for each hand separately, and the data were averaged.

### Sample collection

Blood samples were taken twice: at the beginning and at the end of treatment. Patients’ blood was drawn in 4 mL into vacutainers with the anticoagulant K_2_EDTA. The samples were then transferred to −80 °C for long-term storage (more than 1 week). Phase separation was performed by the standard method. Briefly, plasma and PBMCs were separated from blood cells no later than 30 min after blood collection by centrifugation in a Histopaque density gradient (Sigma, H8889-100ML, USA).

### Monochrome multiplex quantitative PCR telomere length measurement (MMqPCR)

The phenol-chloroform extraction method was used to isolate DNA from leukocytes (Cherska et al., 2020). Leukocyte telomere lengths were determined by the Monochrome Multiplex Quantitative PCR Telomere Length Measurement (MMqPCR) method proposed by Cawthon (2009). The PCR reaction mixture was prepared using a commercial reagent kit (Solis BioDyne HOT FIREPol^®^ Probe qPCR Mix Plus (no ROX), 5 × , Estonia).

The list of primers used for MMqPCR is given in Table 1.

**Table 1:** List of primers for MMqPCR.

Primers	Primer nucleotide sequence
telg	5′-ACACTAAGGTTTGGGTTTGGGTTTGGGTTTGGGTTAGTGT-3′
telc	5′-TGTTAGGTATCCCTATCCCTATCCCTATCCCTATCCCTAACA-3′
albd	5′-GCCCGGCCCGCCGCGCCCGTCCCGCCGGAAAAGCATGGTCGCCTGTT- 3′
albu	5′-CGGCGGCGGGCGGCGCGGGCTGGGCGGAAATGCTGCACAGAATCCTTG-3′

The thermal cycling profile was as follows: 1 cycle 95 °C—15 min; 2 cycles: 95 °C—15 s and 49 °C—15 s; 40 cycles: 95 °C—15 s, 62 °C—10 s, 74 °C—15 s, 84 °C—10 s, 88 °C—15 s; 56 cycles from 70 °C to 5 s in 0.5 °C steps with a signal obtained at each step. Amplification curves were generated by OpticonMonitor 3 software. The relative length of telomeres was calculated as the ratio T/S, where T is the number of telomeric repeats and S is the number of albumin gene repeats (Kolyada et al., 2016; Krasnienkov et al., 2018; Sorochynska et al., 2018; Tronko et al., 2020; Yehorova et al., 2019).

### Mitochondrial DNA copy number, mtDNA-CN

The phenol-chloroform extraction method was used to isolate DNA from leukocytes (Cherska et al., 2020). To determine the number of mtDNA copies (Mitochondrial DNA Copy Number, mtDNA-CN), the MMqPCR method was used. The PCR reaction mixture was prepared using a commercial reagent kit (Solis BioDyne HOT FIREPol® Probe qPCR Mix Plus (no ROX), 5 × , Estonia).

The list of primers used for MMqPCR is given in Table 2.

**Table 2:** List of primers for ММqPCR.

Primers	Primer nucleotide sequence
albd	5′-GCCCGGCCCGCCGCGCCCGTCCCGCCGGAAAAGCATGGTCGCCTGTT-3′
albu	5′-CGGCGGCGGGCGGCGCGGGCTGGGCGGAA ATGCTGCACAGAATCCTTG-3′
D-loop_MPLX_F	5′-ACGCTCGACACACAGCACTTAAACACATCTCTGC-3′
D-loop_MPLX_R	5′-GCTCAGGTCATACAGTATGGGAGTGRGAGGGRAAAA-3′

The thermal cycling profile was as follows: 1 cycle 95 °C—15 min; 2 cycles: 95 °C—15 s and 49 °C—15 s; 40 cycles: 95 °C—15 s, 62 °C—10 s, 74 °C—15 s, 83 °C—10 s, 88 °C—15 s; 66 cycles from 65 °C to 30 s in 0.5 °C steps with a signal obtained at each step. Amplification curves were generated by OpticonMonitor 3 software. The relative number of mtDNA copies was calculated as the M/S ratio, where M is the number of mitochondrial repeats and S is the number of albumin gene repeats (Kolyada et al., 2016; Krasnienkov et al., 2018; Sorochynska et al., 2018; Tronko et al., 2020; Yehorova et al., 2019).

### Measurement of biochemical markers

Protein concentration analysis was performed according to the Bradford method with minor modifications (Marchenko et al., 2024).

LDH activity was determined using a commercial kit (High Technology, Inc., HTI-L7572-120, HTI-L7572-600, HTI-L7572-340, HTI-L7572-540) for PBMCs and plasma. Measurements were performed using a Thermo Scientific Varioskan Flash spectrofluorometer and primarily expressed as U/l. Then obtained values were normalized for total protein content and reported as U/mg of protein or U/mg of protein * 1000 (for PBMCs).

The activity of superoxide dismutase (SOD, EC 1.15.1.1) in blood plasma was determined by an indirect spectrophotometric method based on the reaction of superoxide-dependent oxidation of quercetin in alkaline medium in the presence of tetramethylenediamine (Góth, 1991; Kostyuk and Potapovich, 1989). Measurements were performed using a Thermo Scientific Varioskan Flash spectrofluorometer and primarily expressed as U/ml. The obtained values were normalized to the total protein content of each sample and expressed as arbitrary units per mg of protein.

To determine the activity of blood catalase (EC 1.11.1.6), a method based on the reaction of residual hydrogen peroxide with ammonium molybdate was used (Cherska et al., 2020; Góth, 1991). The measurements were performed using a Thermo Scientific Varioskan Flash spectrofluorometer and expressed in U/ml.

The relative concentration of AGEs in plasma was determined in fluorescence mode (excitation at 355 nm, emission at 440 nm) using a Thermo Scientific Varioskan Flash spectrofluorometer (Dozio et al., 2020; Marchenko et al., 2024). The obtained fluorescence values were normalized to the total protein content of each sample and expressed as arbitrary units per mg of protein (Dozio et al., 2020).

The content of GSH/GSSG in blood plasma in the presence of an imidazole compound was assessed according to the standard method (Marchenko et al., 2024; Mokrasch and Teschke, 1984). The measurements were performed using a Thermo Scientific Varioskan Flash spectrofluorometer and expressed in μM.

The concentration of TBA-active products was measured by heating malondialdehyde (MDA) with 2-thiobarbituric acid (TBA) in an acidic medium to form a colored trimethine complex with a maximum fluorescence emission at *λ* = 530 nm under light excitation from λ = 484 nm (Cherska et al., 2020; Tüközkan et al., 2006). Measurements were performed using a Thermo Scientific Varioskan Flash spectrofluorometer and expressed in μM.

### Statistical analysis

The primary data were processed according to Atramentova (2023). The median (*Me*) and interquartile range (*QI–QIII*) were calculated for all parameters studied. Hypotheses about the effect of the drug were tested using Wilcoxon*’s T*-test (for related samples). To control the false discovery rate (FDR) across all 26 parameters tested, the Benjamini-Hochberg procedure was applied. Differences were considered statistically significant at *α* = 0.05 for both raw *p*-values and BH-adjusted *p*-values (*p_BH_*). Differences for which *p <* 0.05 were considered significant (Atramentova and Utyevska, 2007).

Statistical analysis was performed in Python 3 using the pandas, scipy, numpy, and matplotlib libraries.

## Results

### Clinical and neurophysiological evaluation of the efficacy of EGb 761 treatment in patients with Parkinson’s disease. The unified Parkinson’s disease rating scale (UPDRS) and the Hoehn-Yahr scale

The UPDRS was chosen as a test with a rating scale that is standard in biomedical research and in daily clinical practice to track the dynamics of PD progression in patients with this diagnosis (Abbruzzese et al., 2020). The UPDRS I assesses thinking, behavior and mood; the UPDRS II assesses activities of daily living; and the UPDRS III assesses motor skills. The key is based on a direct correlation between the number of points and the severity of the disease, i.e., as the score increases, the symptoms worsen.

The EGb 761 treatment administration against the background of basic antiparkinsonian therapy did not have a favorable effect on the condition of the majority of the patients tested in UPDRS I.

**Figure 1:**
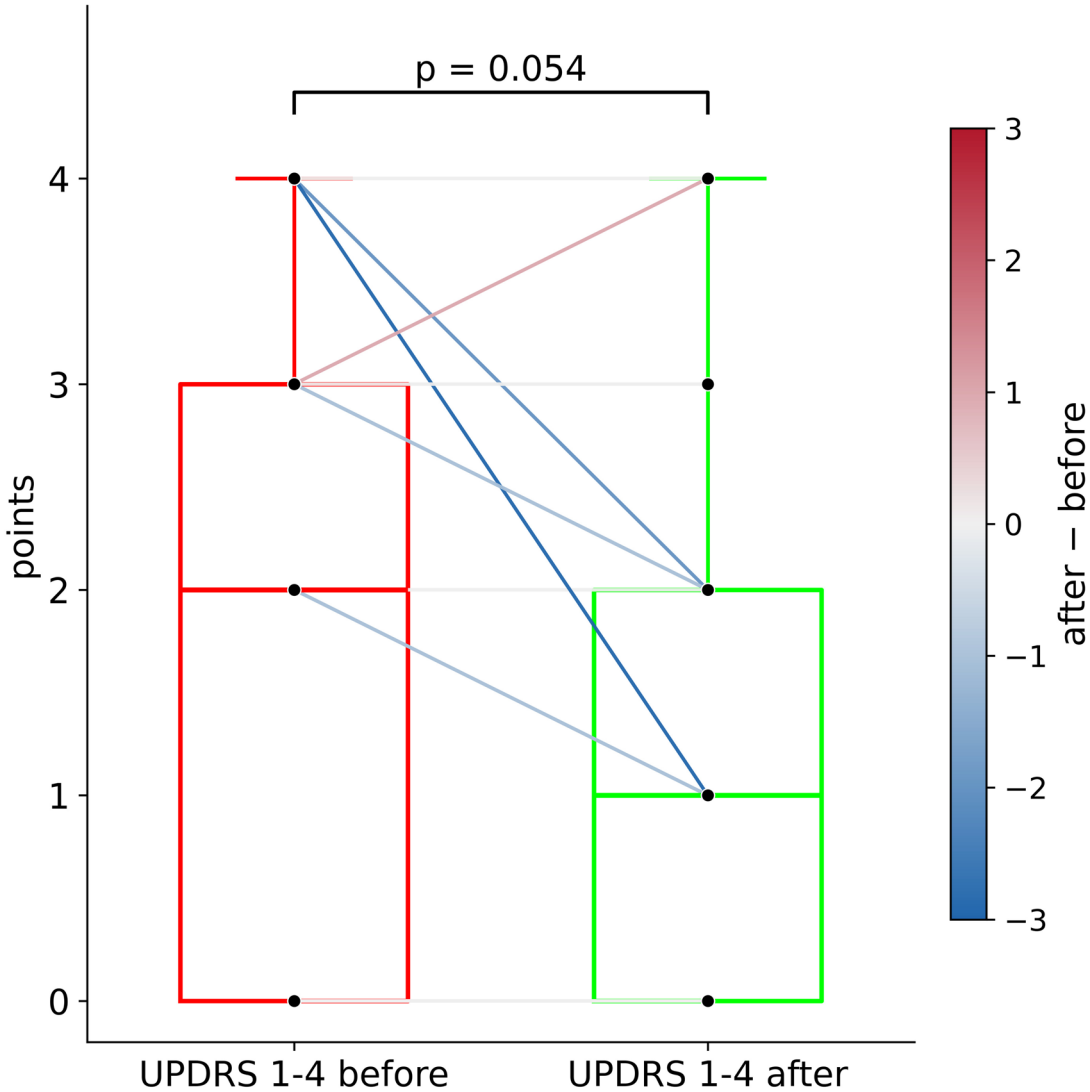
UPDRS I scores before and after the EGb 761 treatment. Boxes with whiskers represent the distribution of results: horizontal lines indicate the first quartile (lower limit of the box), median (middle of the box), and third quartile (upper limit of the box), with whiskers extending to the minimum and maximum values. The dots inside the boxes represent data, and dots corresponding to the same patient are connected by lines, whose color changes on a gradient scale from blue (smallest negative difference) to red (largest positive difference).

The EGb 761 treatment did not lead to statistically significant changes (*p =* 0,054; *p_BH_ =* 0,139) (Figure 1).

Administration of EGb 761 in combination with basic antiparkinsonian therapy had a favorable effect on the condition of the majority of the patients assessed by UPDRS II.

**Figure 2:**
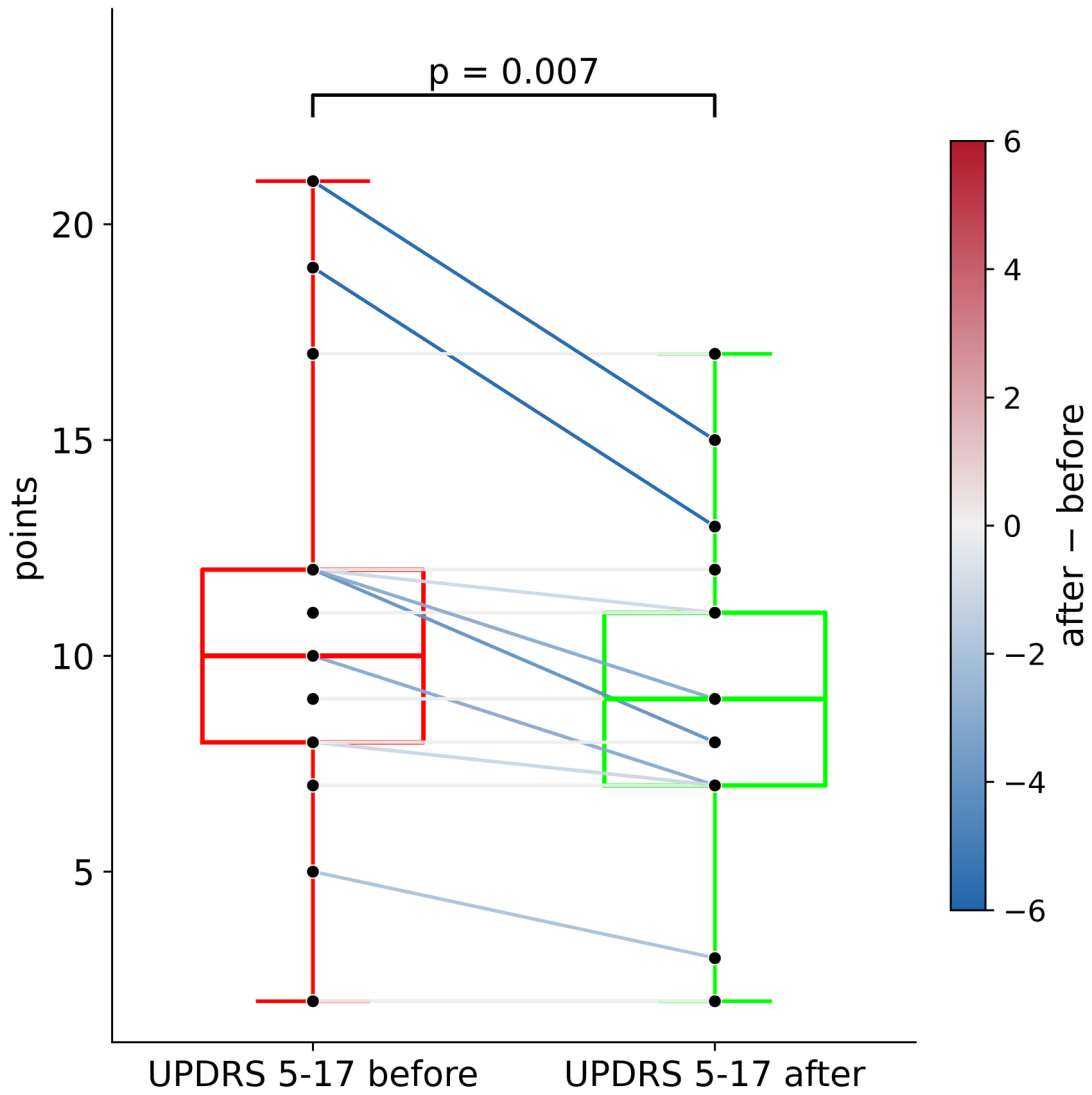
UPDRS II scores before and after the EGb 761 treatment. Boxes with whiskers represent the distribution of results: horizontal lines indicate the first quartile (lower limit of the box), median (middle of the box), and third quartile (upper limit of the box), with whiskers extending to the minimum and maximum values. The dots inside the boxes represent data, and dots corresponding to the same patient are connected by lines, whose color changes on a gradient scale from blue (smallest negative difference) to red (largest positive difference).

The decrease in UPDRS II scores indicates a statistically significant improvement in the quality of basic activities of daily living (*p =* 0,007; *p_BH_ =* 0,039) (Figure 2).

EGb 761 treatment in combination with basic antiparkinsonian therapy had a favorable effect on the condition of the majority of the patients tested in UPDRS III.

**Figure 3:**
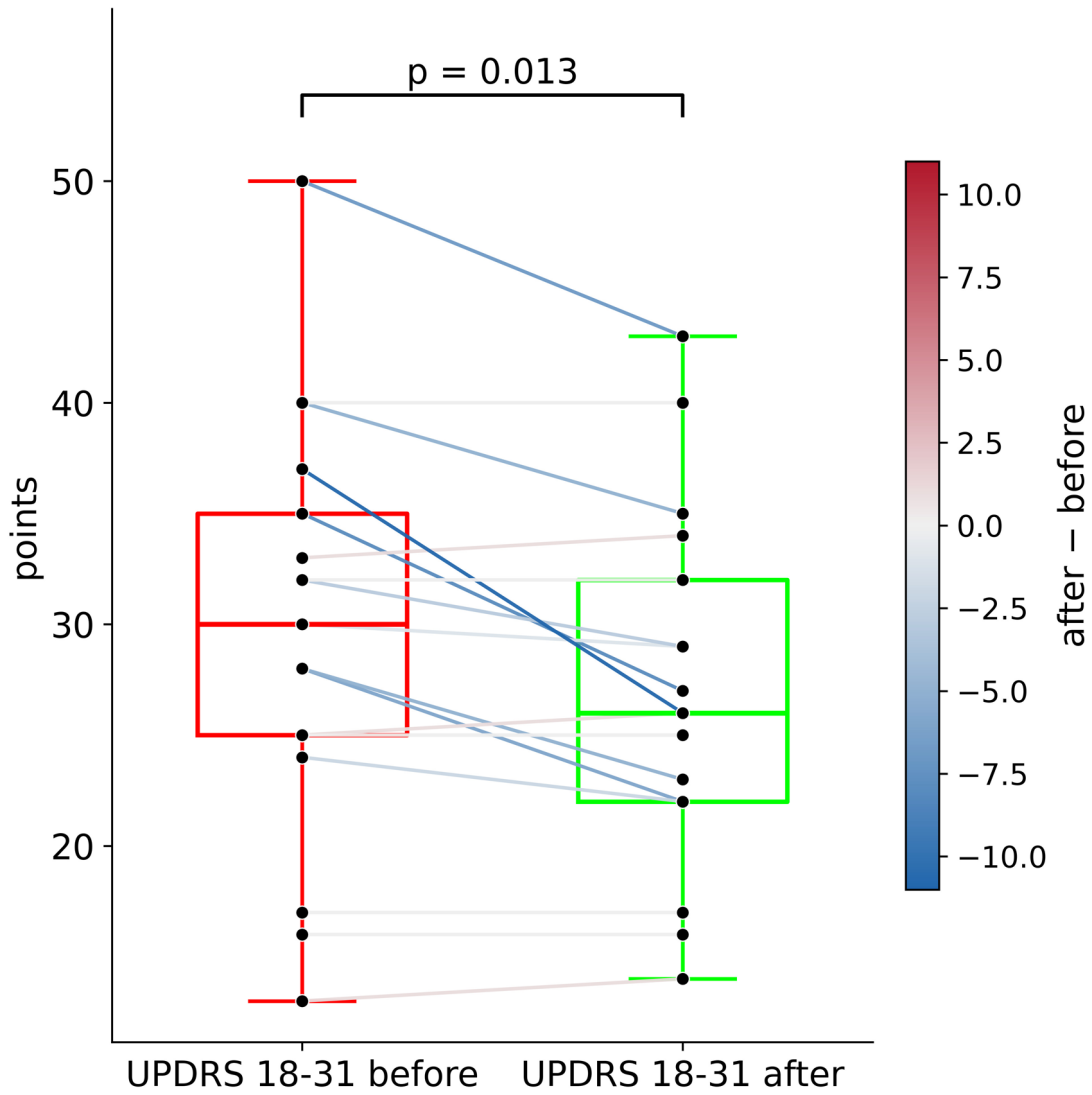
UPDRS III scores before and after the EGb 761 treatment. Boxes with whiskers represent the distribution of results: horizontal lines indicate the first quartile (lower limit of the box), median (middle of the box), and third quartile (upper limit of the box), with whiskers extending to the minimum and maximum values. The dots inside the boxes represent data, and dots corresponding to the same patient are connected by lines, whose color changes on a gradient scale from blue (smallest negative difference) to red (largest positive difference).

The decrease in UPDRS III scores indicates a statistically significant improvement in the quality of patients’ motor skills (*p =* 0,013; *p_BH_ =* 0,049) (Figure 3).

Patients showed a statistically significant improvement in total UPDRS scores.

**Figure 4:**
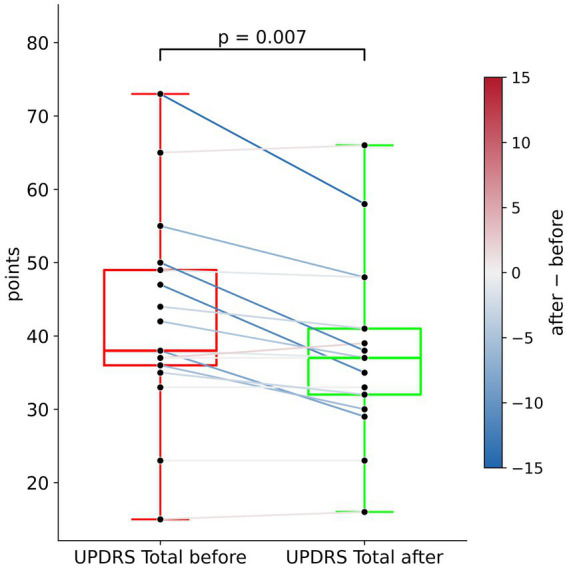
UPDRS scores before and after the EGb 761 treatment. Boxes with whiskers represent the distribution of results: horizontal lines indicate the first quartile (lower limit of the box), median (middle of the box), and third quartile (upper limit of the box), with whiskers extending to the minimum and maximum values. The dots inside the boxes represent data, and dots corresponding to the same patient are connected by lines, whose color changes on a gradient scale from blue (smallest negative difference) to red (largest positive difference).

The decrease in UPDRS scores indicates a statistically significant improvement in the condition of patients (*p =* 0,007; *p_BH_ =* 0,039) (Figure 4).

Thus, a month-long extended therapy with EGb 761 statistically significantly improved UPDRS II (daily living), UPDRS III (motor skills), and total UPDRS scores in patients with Parkinson’s disease, along with a trend toward improvement in UPDRS I (thinking, behavior, and mood).

**Figure 5:**
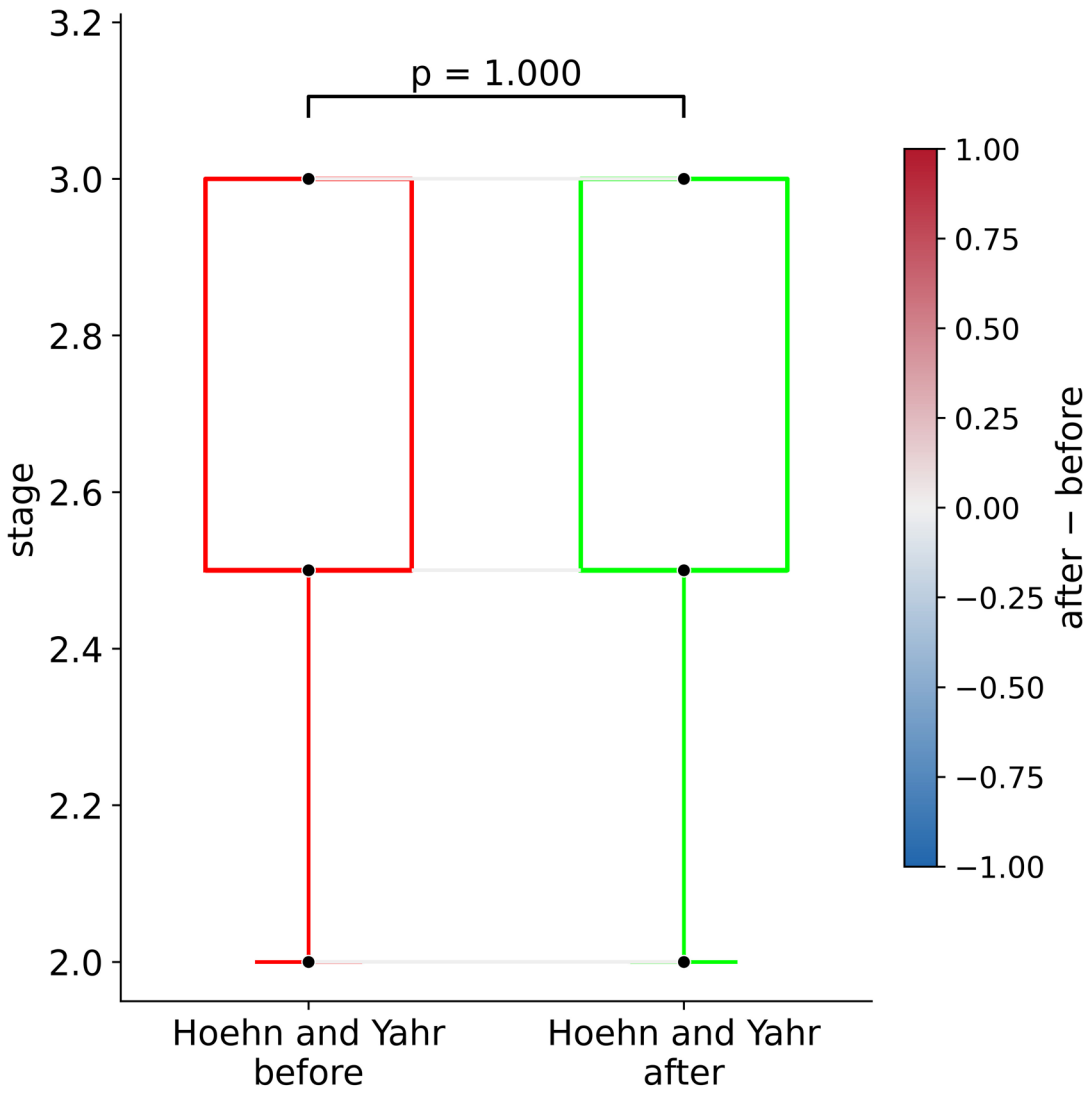
Hoehn and Yahr scale scores before and after the EGb 761 treatment. Boxes with whiskers represent the distribution of results: horizontal lines indicate the first quartile (lower limit of the box), median (middle of the box), and third quartile (upper limit of the box), with whiskers extending to the minimum and maximum values. The dots inside the boxes represent data, and dots corresponding to the same patient are connected by lines, whose color changes on a gradient scale from blue (smallest negative difference) to red (largest positive difference).

Characteristics of PD stages using the Hoehn and Yahr scale showed no changes in any clinical case (Figure 5).

### Mini-Mental State Examination (MMSE)

The assessment of the cognitive profile of patients in clinical practice is carried out mainly with the help of two instruments, one of which is the Mini Mental State Examination (MMSE). The MMSE is a commonly used test for detecting cognitive dysfunction in patients diagnosed with PD (Yu et al., 2020; Chan et al., 2022).

The MMSE takes 5–10 min to complete (Scheffels et al., 2020; Breder et al., 2017). The key contains a 30-point scale. Individuals with Mild Cognitive Impairment (MCI) frequently score between 24 and 29 on the MMSE, and higher scores are interpreted as normal. Decreasing MMSE scores reflect the onset of cognitive impairment and PDD (Parkinson’s disease dementia), making this test a useful clinical indicator of disease progression, as most subjects with PD who did not have dementia at baseline will have a normal MMSE score (Chan et al., 2022).

The MMSE, compared to the MoCA, another cognitive assessment system, can better track cognitive changes over time (Burdick et al., 2014). However, it is more difficult to detect mild cognitive impairment (Koshimoto et al., 2023).

The administration of EGb 761 in combination with basic antiparkinsonian therapy had a favorable effect on the condition of the majority of the patients tested by MMSE.

**Figure 6:**
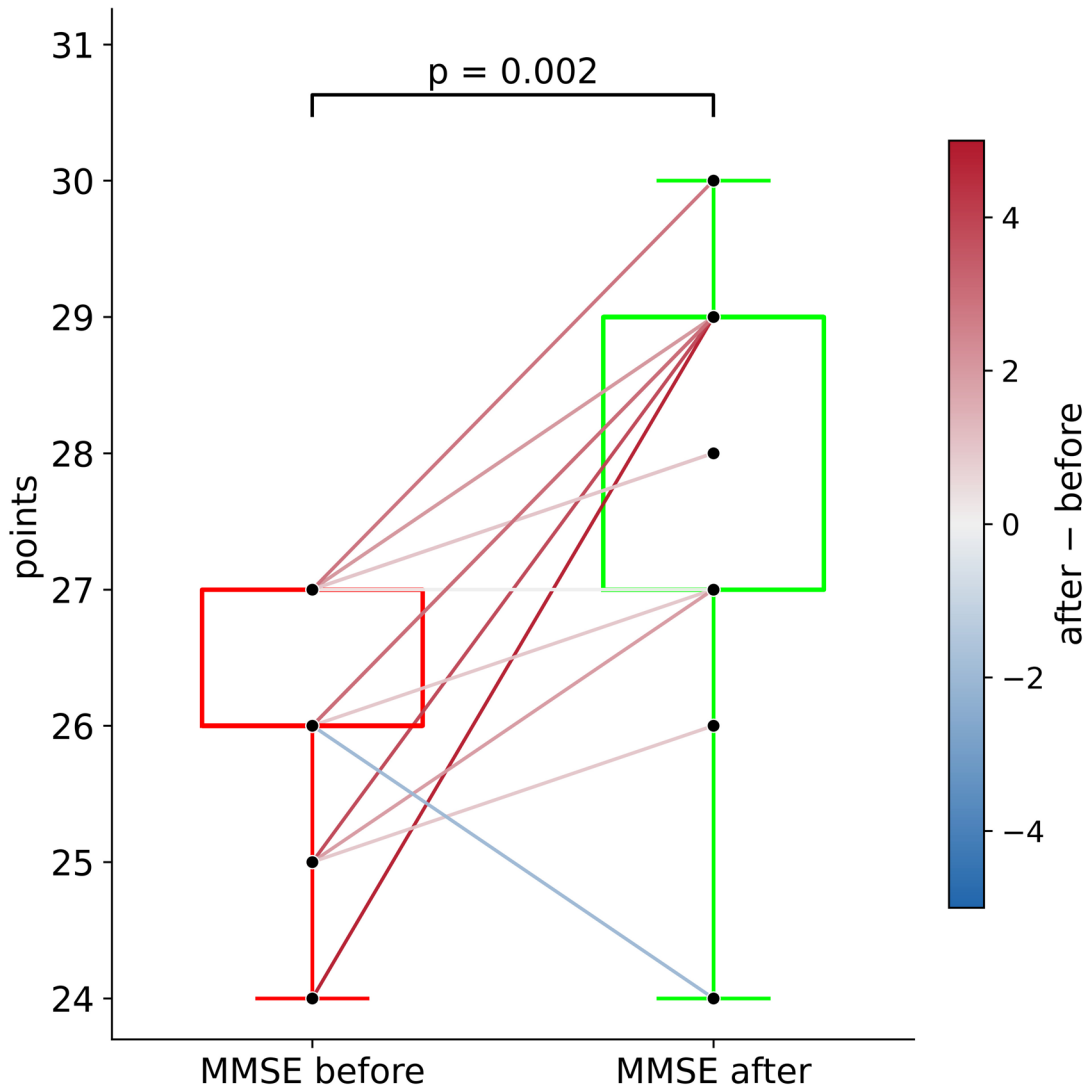
MMSE scores before and after the EGb 761 treatment. Boxes with whiskers represent the distribution of results: horizontal lines indicate the first quartile (lower limit of the box), median (middle of the box), and third quartile (upper limit of the box), with whiskers extending to the minimum and maximum values. The dots inside the boxes represent data, and dots corresponding to the same patient are connected by lines, whose color changes on a gradient scale from blue (smallest negative difference) to red (largest positive difference).

EGb 761 treatment combined with basic antiparkinsonian therapy resulted in statistically significant changes (*p =* 0,002; *p_BH_ =* 0,033) (Figure 6).

### Montreal Cognitive Assessment (MoCA)

The Montreal Cognitive Assessment (MoCA) is a test of cognitive functions. Compared to the MMSE, it is less susceptible to floor and ceiling effects and has greater sensitivity in detecting executive dysfunctions (Yu et al., 2020; Breder et al., 2017). At the same time, it allows for a more reliable detection of mild cognitive impairment compared to the MMSE (Koshimoto et al., 2023). The MoCA is effective regardless of the cultural characteristics of patients (Breder et al., 2017).

The MoCA takes 10 min to complete (Scheffels et al., 2020; Breder et al., 2017). The key contains a 30-point scale, with a threshold score of 26 points or more interpreted as age-equivalent performance (Scheffels et al., 2020).

EGb 761 treatment in combination with basic antiparkinsonian therapy had a favorable effect on the condition of the majority of the patients assessed by MoCA.

**Figure 7:**
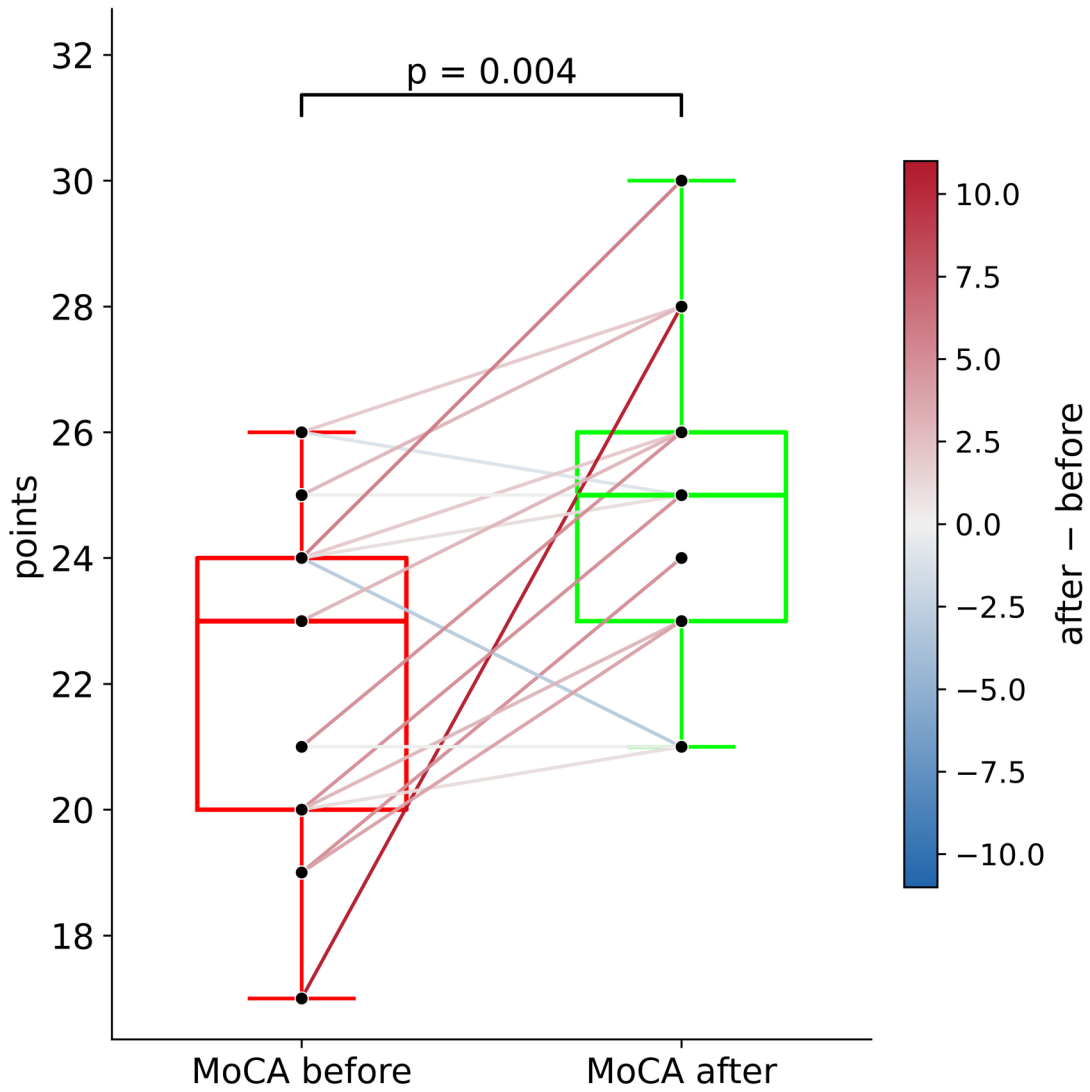
MoCA scores before and after the EGb 761 treatment. Boxes with whiskers represent the distribution of results: horizontal lines indicate the first quartile (lower limit of the box), median (middle of the box), and third quartile (upper limit of the box), with whiskers extending to the minimum and maximum values. The dots inside the boxes represent data, and dots corresponding to the same patient are connected by lines, whose color changes on a gradient scale from blue (smallest negative difference) to red (largest positive difference).

The effect of EGb761 on the state of cognitive functions showed a statistically significant improvement in the MoCA test (*p =* 0,004; *p_BH_ =* 0,034) (Figure 7).

### The Frontal Assessment Battery (FAB) test battery for the assessment of frontal dysfunction

The Test battery for assessing frontal dysfunction or Frontal Assessment Battery (FAB) is a test for assessing executive functions in the diagnosis of neurodegenerative and other diseases affecting the frontostriatal brain circuits (Fernández-Fleites et al., 2021; Grandi et al., 2022).

The FAB was developed for the rapid assessment of executive functions and consists of six tasks that assess thinking, conceptualization, inhibitory control, motor programming, resistance to interference, and autonomy in the environment (Fernández-Fleites et al., 2021; Alster et al., 2021).

FAB takes 10 min to complete (Alster et al., 2021; Han et al., 2020). None of the tasks requires additional tools and does not require patients to perform complex movements (Alster et al., 2021). The key contains an 18-point scale. Each subtest is scored with a maximum of 3 points, and the maximum total score corresponds to the preservation of executive functions (Fernández-Fleites et al., 2021).

Treatment with EGb 761 in combination with basic antiparkinsonian therapy did not have a favorable effect on the condition of the majority of the patients evaluated using FAB.

**Figure 8:**
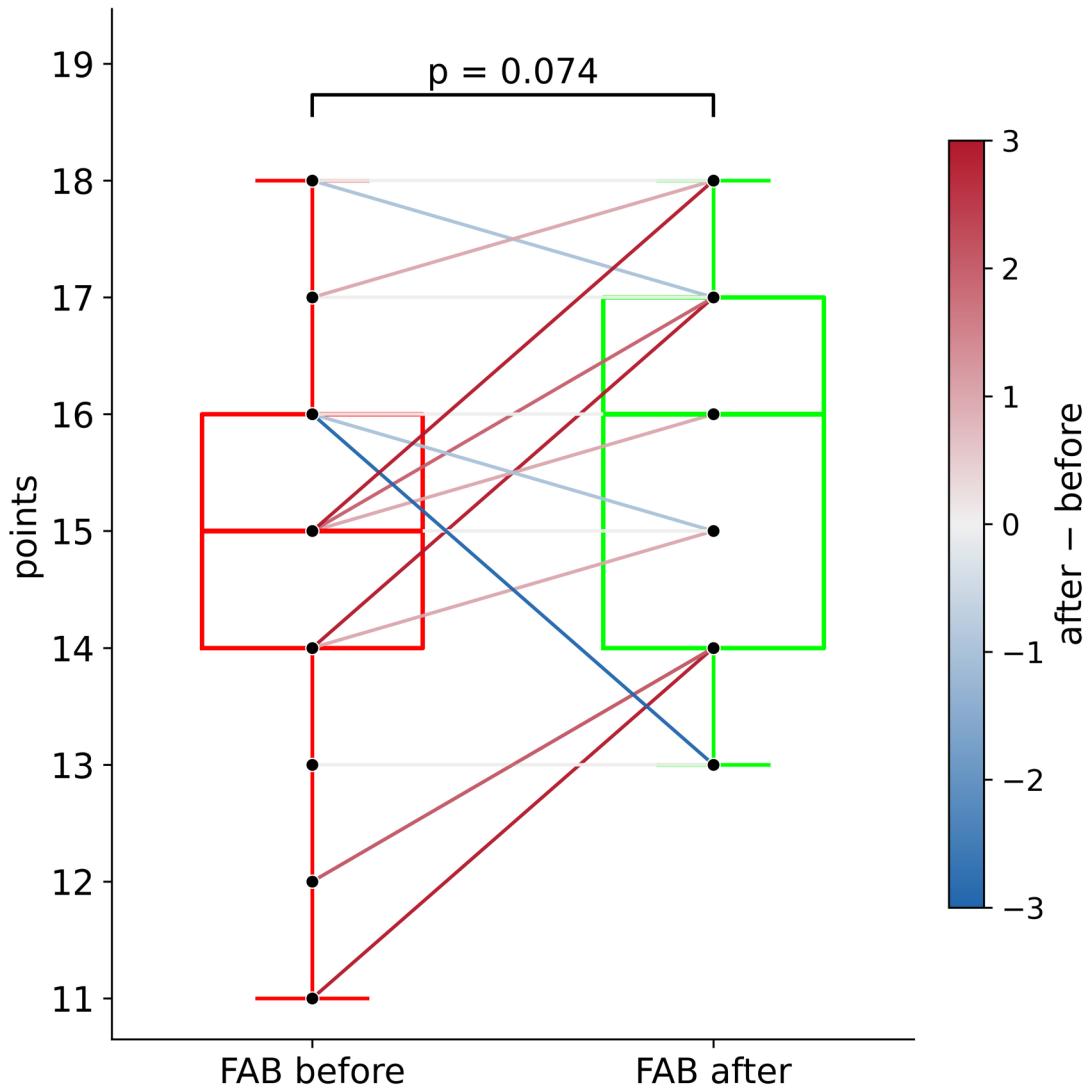
FAB scores before and after the EGb 761 treatment. Boxes with whiskers represent the distribution of results: horizontal lines indicate the first quartile (lower limit of the box), median (middle of the box), and third quartile (upper limit of the box), with whiskers extending to the minimum and maximum values. The dots inside the boxes represent data, and dots corresponding to the same patient are connected by lines, whose color changes on a gradient scale from blue (smallest negative difference) to red (largest positive difference).

The effect of EGb761 on the state of cognitive functions did not show a statistically significant improvement in the results of the FAB test (*p =* 0,074; *p_BH_ =* 0,174) (Figure 8).

### Beck Depression Inventory (BDI-I)

The standard clinical test for depression is the Beck Depression Inventory (BDI-I), which has a key that increases the severity of depression as the score increases. When the test is scored, each response is assigned a value between 0 and 3, and the sum of the scores is then compared to the key to determine the severity of depression. The standard grade boundaries are as follows: 1–10 for normal, 11–16 for mild mood disorder, 17–20 for borderline clinical depression, 21–30 for moderate depression, 31–40 for severe depression, and >40 for extreme depression (Saha et al., 2019).

Treatment with EGb 761 in combination with basic antiparkinsonian therapy did not have a favorable effect on the emotional and motivational state of the majority of the patients assessed by the Beck method.

**Figure 9:**
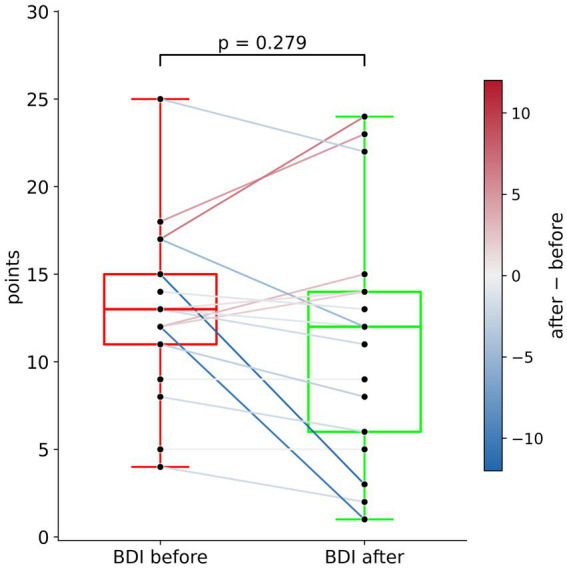
BDI-I scores before and after the EGb 761 treatment. Boxes with whiskers represent the distribution of results: horizontal lines indicate the first quartile (lower limit of the box), median (middle of the box), and third quartile (upper limit of the box), with whiskers extending to the minimum and maximum values. The dots inside the boxes represent data, and dots corresponding to the same patient are connected by lines, whose color changes on a gradient scale from blue (smallest negative difference) to red (largest positive difference).

The effect of EGb761 on Beck Depression Scale scores did not show a statistically significant improvement in the BDI-I test results (*p =* 0,279; *p_BH_ =* 0,483) (Figure 9).

### STAI

The non-motor symptoms of PD include increased anxiety, which is recorded in 12–57% of patients with PD (Yang et al., 2019; Rutten et al., 2017). It often precedes the development of motor symptoms (Rutten et al., 2017), and over time, it begins to accompany symptoms of depression, which worsens quality of life and has a devastating impact on social and occupational functioning (Hanna and Cronin-Golomb, 2012).

Treatment with EGb 761 in combination with basic antiparkinsonian therapy did not have a favorable effect on the emotional and motivational state of the majority of the patients assessed by the STAI methodology, either in the reactive anxiety or personal anxiety subscales.

**Figure 10:**
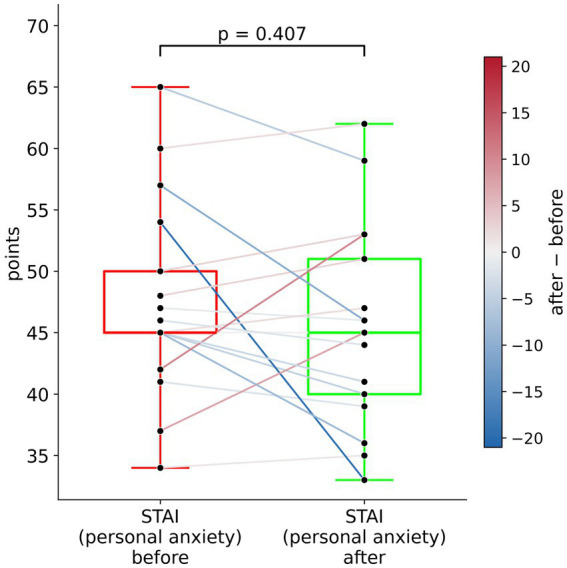
STAI (reactive anxiety) (left) and STAI (personal anxiety) (right) scores before and after the EGb 761 treatment. Boxes with whiskers represent the distribution of results: horizontal lines indicate the first quartile (lower limit of the box), median (middle of the box), and third quartile (upper limit of the box), with whiskers extending to the minimum and maximum values. The dots inside the boxes represent data, and dots corresponding to the same patient are connected by lines, whose color changes on a gradient scale from blue (smallest negative difference) to red (largest positive difference).

The effect of EGb761 on depression scores on the STAI scale did not show a statistically significant improvement in the results of the STAI (reactive anxiety) (*p =* 0,153; *p_BH_ =* 0,283) test and STAI (personal anxiety) test (*p =* 0,407; *p_BH_ =* 0,588) (Figure 10).

### Motor tempo

The Motor Tempo Test (MT) was chosen for instrumental assessment of the effect of the drug on motor functions. This test involves checking the speed and coordination of movements.

Treatment with EGb 761 in combination with basic antiparkinsonian therapy did not have a favorable effect on the motor function of patients assessed by MT.

**Figure 11:**
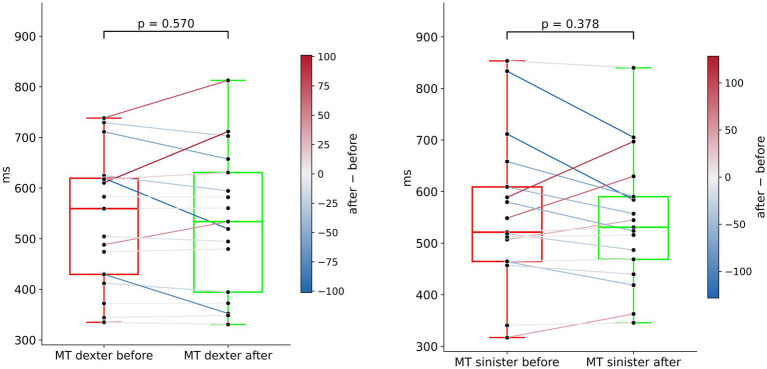
MT scores before and after EGb 761 for the left hand (left) and right hand (right). Boxes with whiskers represent the distribution of results: horizontal lines indicate the first quartile (lower limit of the box), median (middle of the box), and third quartile (upper limit of the box), with whiskers extending to the minimum and maximum values. The dots inside the boxes represent data, and dots corresponding to the same patient are connected by lines, whose color changes on a gradient scale from blue (smallest negative difference) to red (largest positive difference).

The effect of EGb761 on MT scores did not show a statistically significant improvement in either the left arm (*p =* 0,378; *p_BH_ =* 0,588) or the right arm (*p =* 0,570; *p_BH_ =* 0,741) (Figure 11).

### Sensorimotor response

The Sensorimotor reaction (SMR) test was chosen to assess the effect of the drug on motor functions. This test involves checking not so much the speed and coordination of movements, as in the Motor tempo test, but rather the processes of analyzing sensory information and making decisions.

EGb 761 treatment in combination with basic antiparkinsonian therapy had a favorable effect on the motor function of the majority of the patients assessed by SMR.

**Figure 12:**
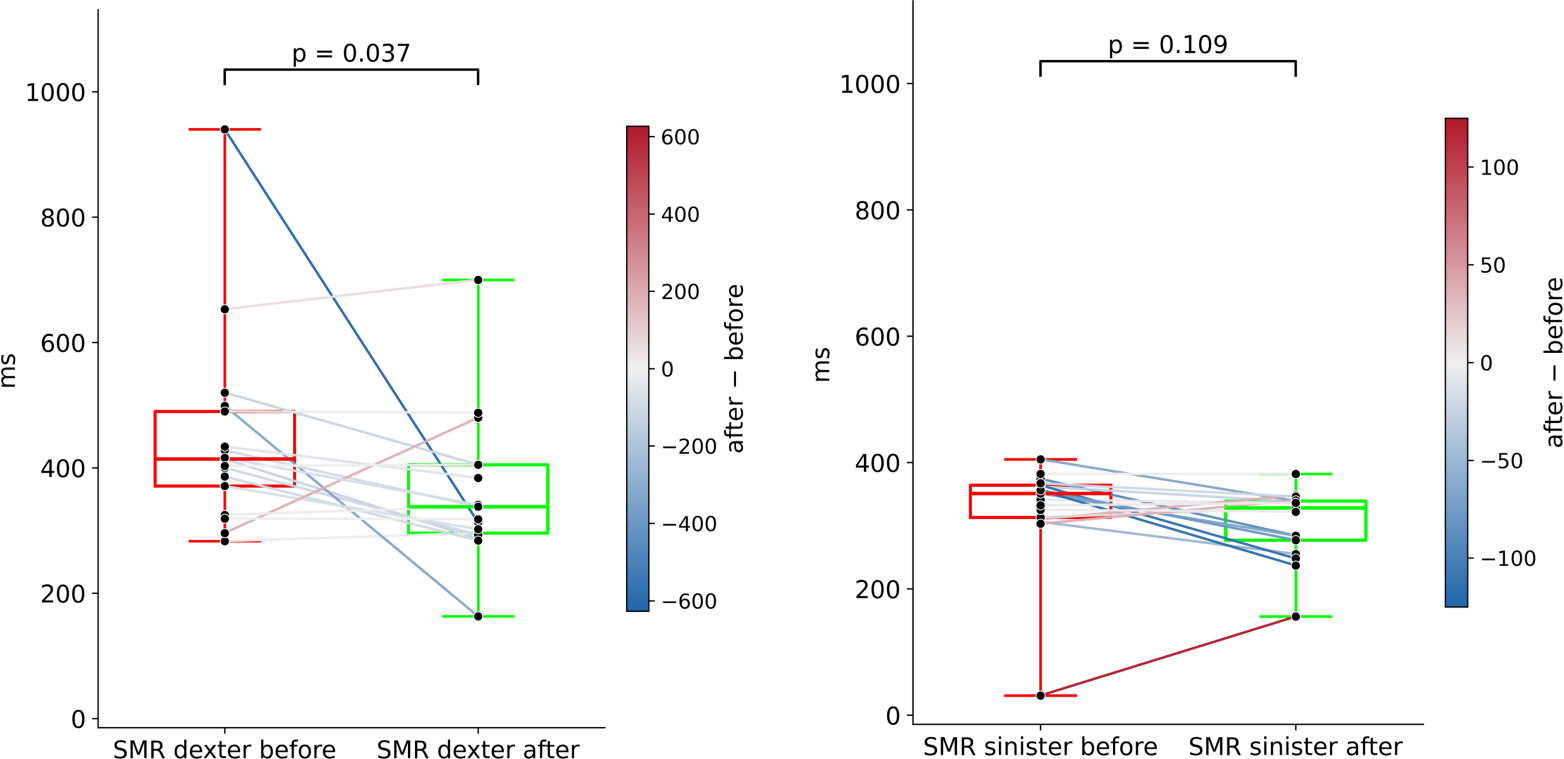
SMR values before and after EGb 761 for the left hand (left) and right hand (right). Boxes with whiskers represent the distribution of results: horizontal lines indicate the first quartile (lower limit of the box), median (middle of the box), and third quartile (upper limit of the box), with whiskers extending to the minimum and maximum values. The dots inside the boxes represent data, and dots corresponding to the same patient are connected by lines, whose color changes on a gradient scale from blue (smallest negative difference) to red (largest positive difference).

These changes are mainly characteristic of the reactions of the right hand (*p =* 0,037; *p_BH_ =* 0,114), for the left hand the changes are not statistically significant (*p =* 0,109; *p_BH_ =* 0,236) (Figure 12).

### Relative average telomere length

**Figure 13:**
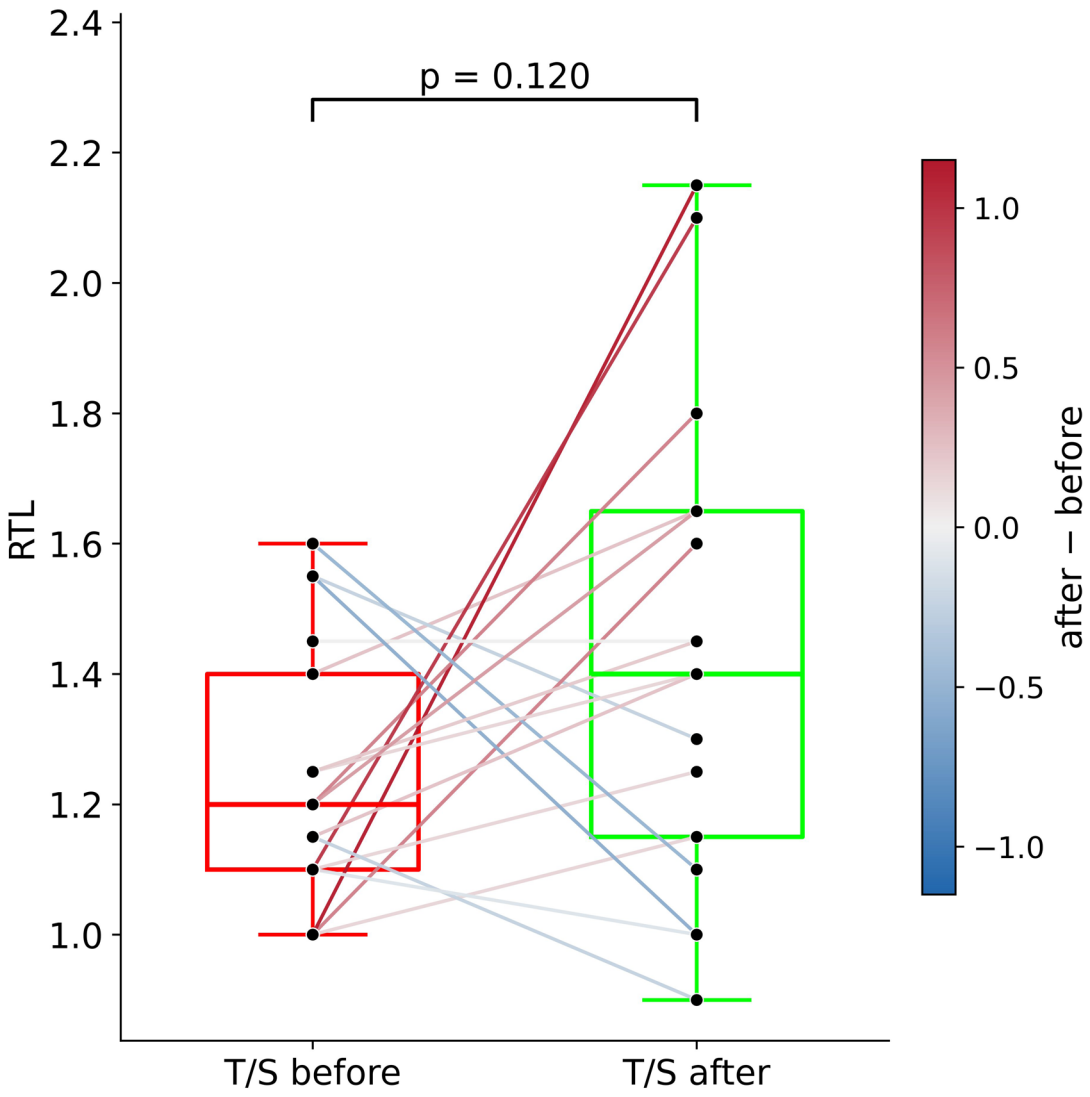
RTL scores before and after the EGb 761 treatment. Boxes with whiskers represent the distribution of results: horizontal lines indicate the first quartile (lower limit of the box), median (middle of the box), and third quartile (upper limit of the box), with whiskers extending to the minimum and maximum values. The dots inside the boxes represent data, and dots corresponding to the same patient are connected by lines, whose color changes on a gradient scale from blue (smallest negative difference) to red (largest positive difference).

Adjunctive therapy did not lead to statistically significant changes in the relative length of leukocyte telomeres. No significant changes in the relative lengths of telomeres were detected, only a tendency to their increase was observed (*p =* 0,120; *p_BH_ =* 0,241) (Figure 13).

### Relative number of mtDNA copies

**Figure 14:**
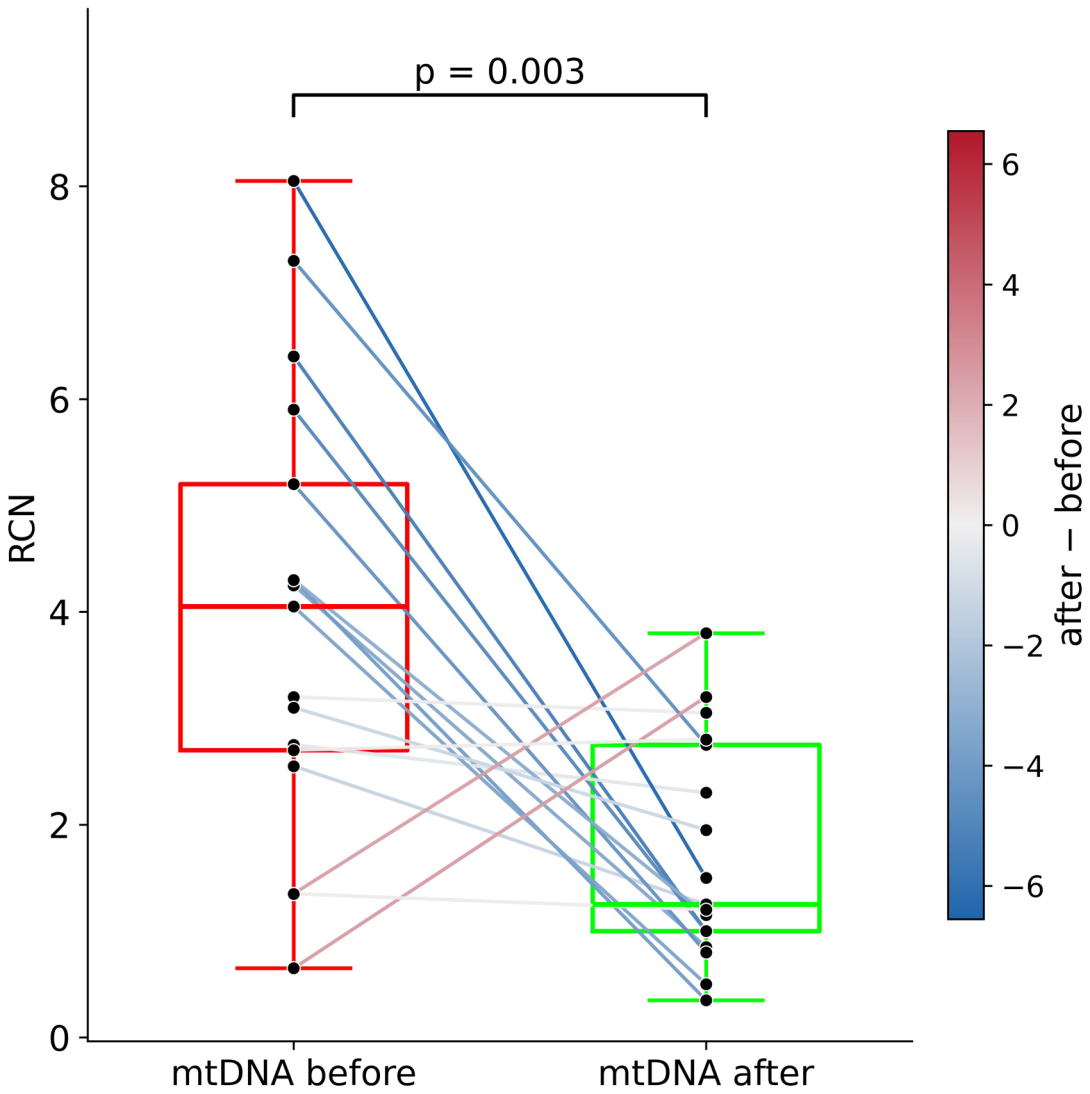
RCN scores before and after the EGb 761 treatment. Boxes with whiskers represent the distribution of results: horizontal lines indicate the first quartile (lower limit of the box), median (middle of the box), and third quartile (upper limit of the box), with whiskers extending to the minimum and maximum values. The dots inside the boxes represent data, and dots corresponding to the same patient are connected by lines, whose color changes on a gradient scale from blue (smallest negative difference) to red (largest positive difference).

EGb 761 treatment in combination with basic antiparkinsonian therapy did not have a favorable effect on the relative mtDNA numbers in the majority of the patients. Significant changes were observed in the relative number of mtDNA copies (*p =* 0,003; *p_BH_ =* 0,033) (Figure 14).

### Markers of oxidative stress

Adjunctive therapy with the addition of EGb 761 did not lead to changes in SOD activity.

**Figure 15:**
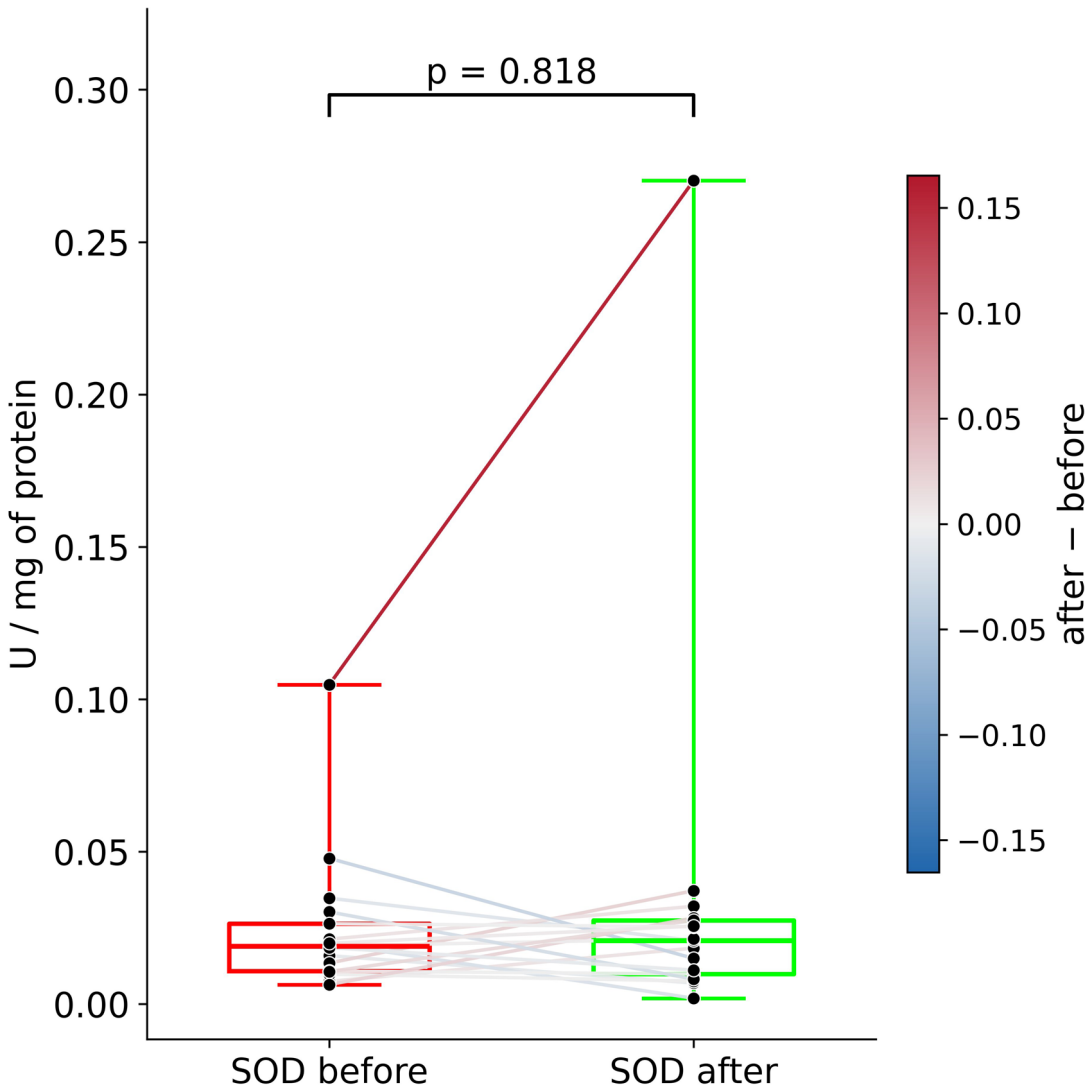
Indicators of SOD activity before and after EGb 761 treatment. Boxes with whiskers represent the distribution of results: horizontal lines indicate the first quartile (lower limit of the box), median (middle of the box), and third quartile (upper limit of the box), with whiskers extending to the minimum and maximum values. The dots inside the boxes represent data, and dots corresponding to the same patient are connected by lines, whose color changes on a gradient scale from blue (smallest negative difference) to red (largest positive difference).

EGb 761 treatment in combination with basic antiparkinsonian therapy did not lead to statistically significant changes (*p =* 0,818; *p_BH_ =* 0,942) in SOD activity (Figure 15).

EGb 761 treatment in combination with basic antiparkinsonian therapy did not affect CAT activity.

**Figure 16:**
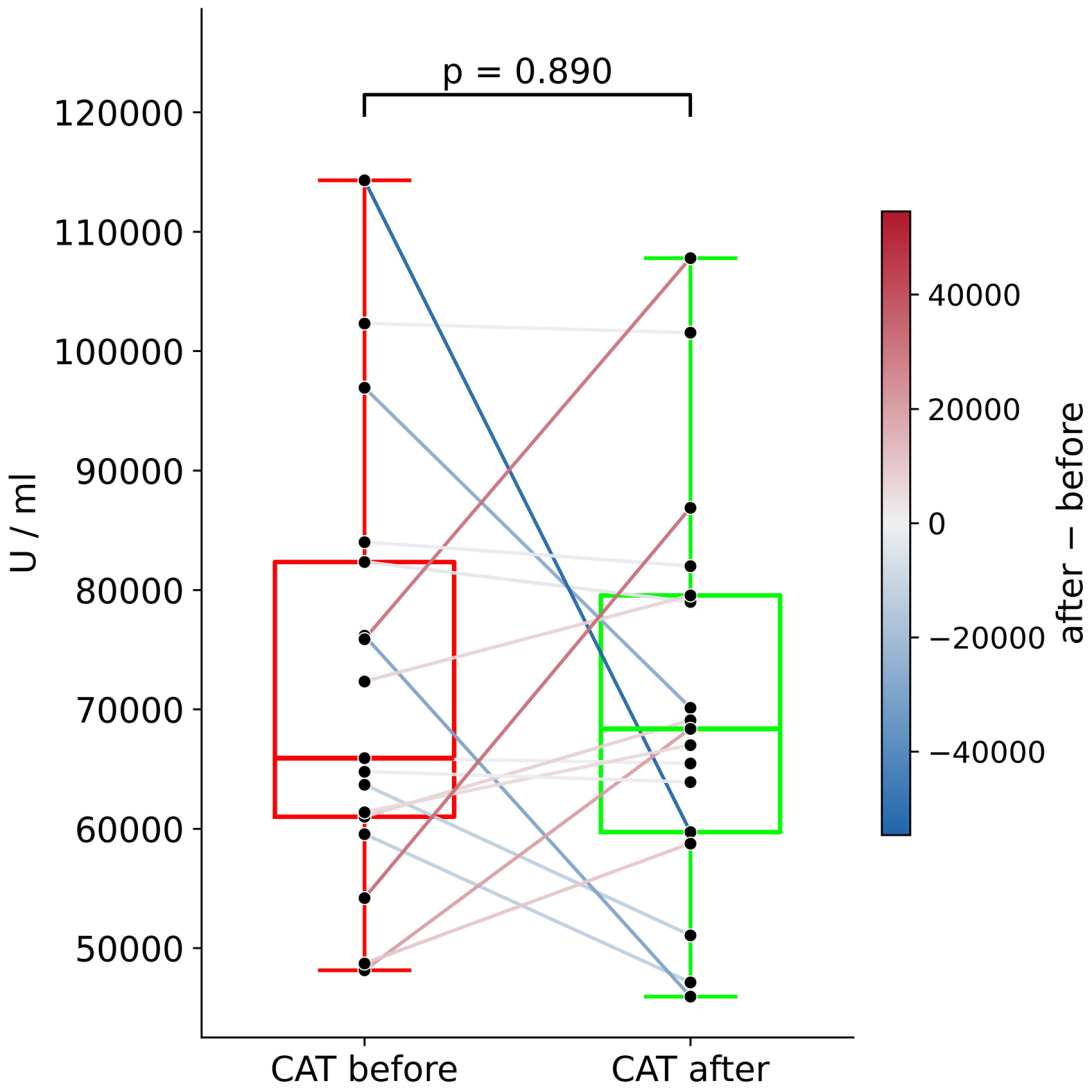
CAT activity before and after the EGb 761 treatment. Boxes with whiskers represent the distribution of results: horizontal lines indicate the first quartile (lower limit of the box), median (middle of the box), and third quartile (upper limit of the box), with whiskers extending to the minimum and maximum values. The dots inside the boxes represent data, and dots corresponding to the same patient are connected by lines, whose color changes on a gradient scale from blue (smallest negative difference) to red (largest positive difference).

EGb 761 treatment in combination with basic antiparkinsonian therapy did not lead to statistically significant changes in CAT activity (*p =* 0,890; *p_BH_ =* 0,942) (Figure 16).

Adjunctive therapy with the addition of EGb 761 did not lead to statistically significant changes in the relative concentration of AGEs.

**Figure 17:**
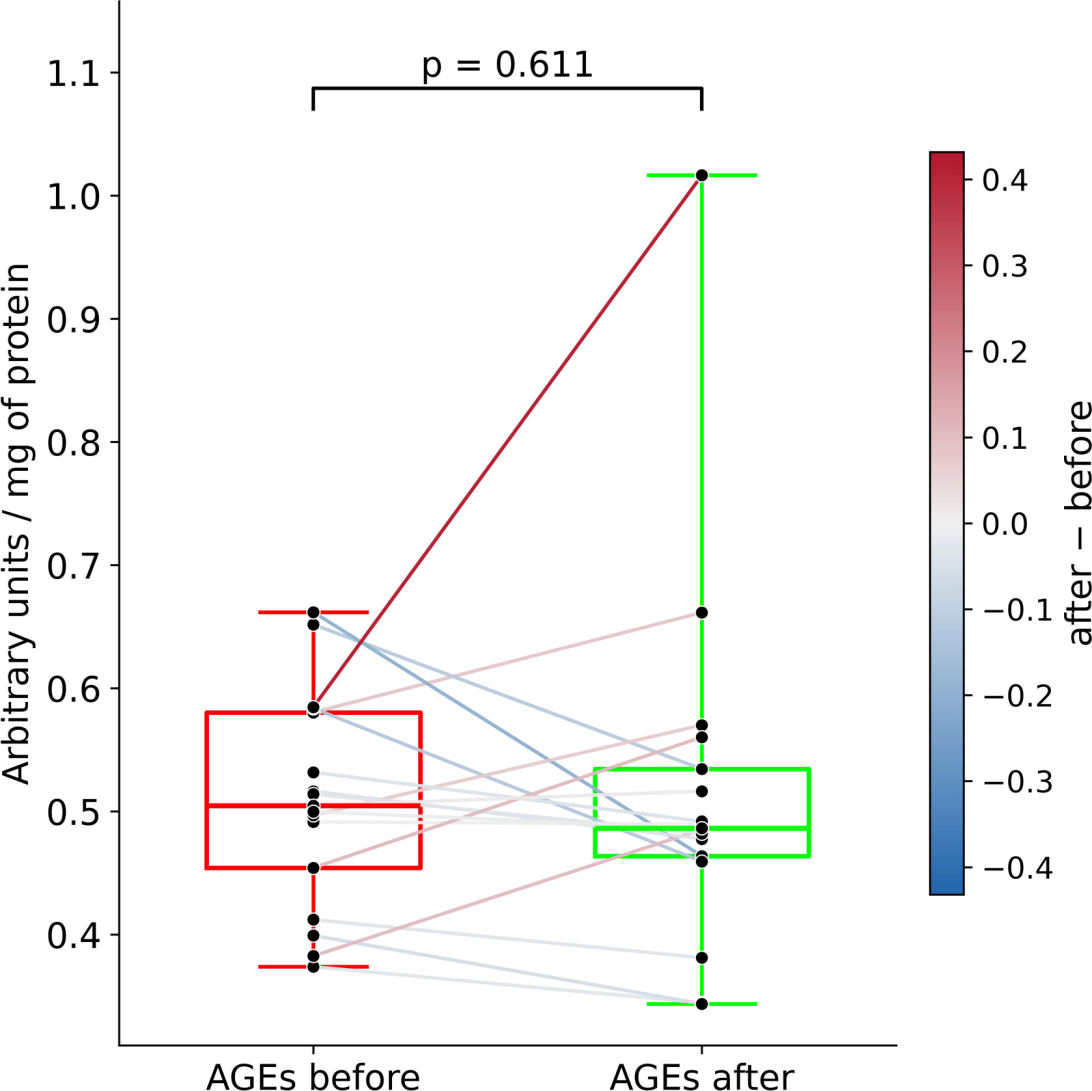
Level of AGEs before and after the EGb 761 treatment. Boxes with whiskers represent the distribution of results: horizontal lines indicate the first quartile (lower limit of the box), median (middle of the box), and third quartile (upper limit of the box), with whiskers extending to the minimum and maximum values. The dots inside the boxes represent data, and dots corresponding to the same patient are connected by lines, whose color changes on a gradient scale from blue (smallest negative difference) to red (largest positive difference).

EGb 761 treatment in combination with basic antiparkinsonian therapy did not lead to statistically significant changes in the levels of AGEs (*p =* 0,611; *p_BH_ =* 0,757) (Figure 17).

EGb 761 treatment in combination with basic antiparkinsonian therapy affected the concentration of GSH/GSSG, and the concentration of one of the key low-molecular-weight antioxidants decreased.

**Figure 18:**
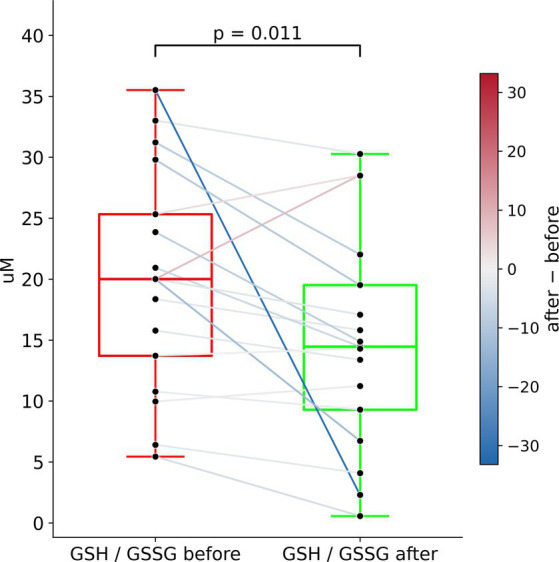
GSH/GSSG concentration before and after the EGb 761 treatment. Boxes with whiskers represent the distribution of results: horizontal lines indicate the first quartile (lower limit of the box), median (middle of the box), and third quartile (upper limit of the box), with whiskers extending to the minimum and maximum values. The dots inside the boxes represent data, and dots corresponding to the same patient are connected by lines, whose color changes on a gradient scale from blue (smallest negative difference) to red (largest positive difference).

EGb 761 treatment in combination with basic antiparkinsonian therapy led to statistically significant changes in GSH/GSSG concentration (*p =* 0,011; *p_BH_ =* 0,048) (Figure 18).

EGb 761 treatment in combination with basic antiparkinsonian therapy affected the concentration of MDA and other TBARS, and the concentration of one of the key markers of oxidative stress increased.

**Figure 19:**
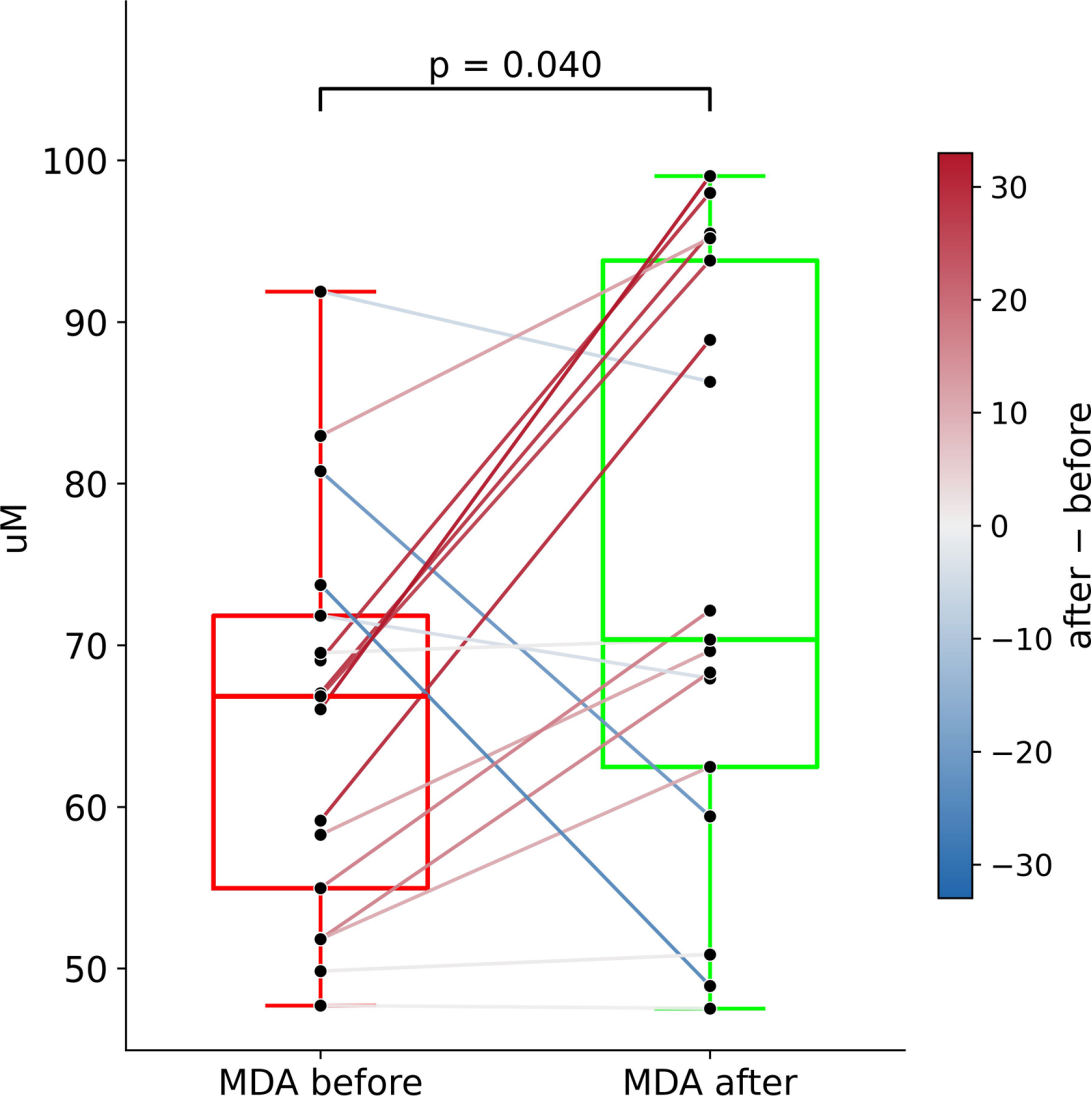
MDA concentration before and after EGb 761 treatment. Boxes with whiskers represent the distribution of results: horizontal lines indicate the first quartile (lower limit of the box), median (middle of the box), and third quartile (upper limit of the box), with whiskers extending to the minimum and maximum values. The dots inside the boxes represent data, and dots corresponding to the same patient are connected by lines, whose color changes on a gradient scale from blue (smallest negative difference) to red (largest positive difference).

EGb 761 treatment in combination with basic antiparkinsonian therapy led to statistically significant changes in MDA concentration (*p =* 0,039; *p_BH_ =* 0,114) (Figure 19).

### Energy metabolism

Adjunctive therapy with the addition of EGb 761 did not lead to statistically significant changes in LDH activity in both PBMCs and plasma.

**Figure 20:**
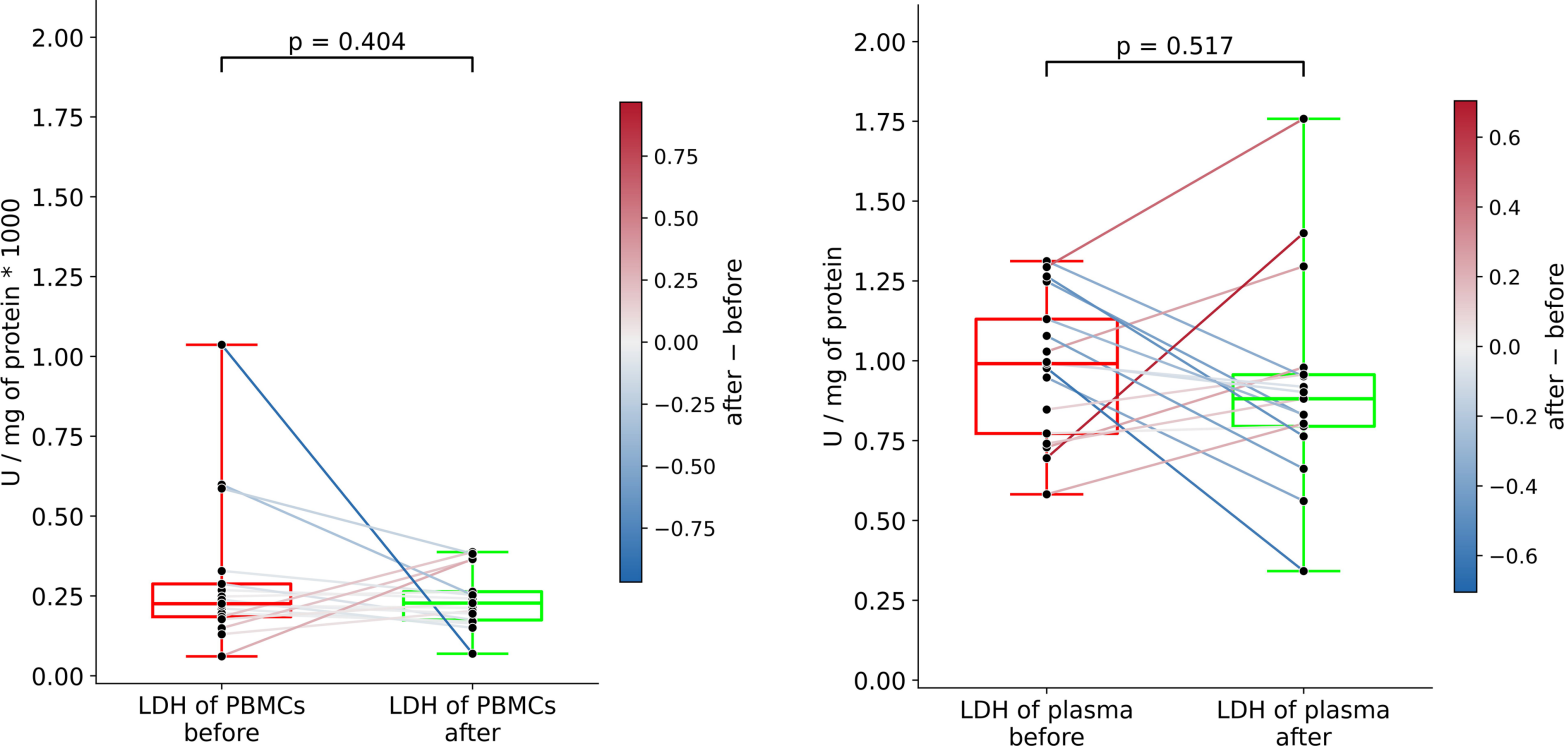
LDH activity in PBMCs (left) and plasma (right) before and after adjunctive therapy. Boxes with whiskers represent the distribution of results: horizontal lines indicate the first quartile (lower limit of the box), median (middle of the box), and third quartile (upper limit of the box), with whiskers extending to the minimum and maximum values. The dots inside the boxes represent data, and dots corresponding to the same patient are connected by lines, whose color changes on a gradient scale from blue (smallest negative difference) to red (largest positive difference).

EGb 761 treatment in combination with basic antiparkinsonian therapy did not lead to statistically significant changes in LDH activity in PBMCs (*p =* 0,404; *p_BH_ =* 0,588) and plasma (*p =* 0,517; *p_BH_ =* 0,708) (Figure 20).

## Discussion

Our study showed that one-month EGb 761 treatment combined with basic antiparkinsonian therapy was associated with improved scores on two clinical and neurophysiological tests, the MMSE and MoCA. This observation suggests that an EGb 761 treatment may enhance cognitive abilities of patients. Simultaneously, UPDRS I, BDI-I and STAI scores for thinking, behavior and mood showed no differences before and after the adjunctive therapy course. Regarding executive functioning, no significant differences were observed in FAB scores.

However, it should be noted that, while the presented tests can be useful for detecting cognitive impairment in PD, their general methodological significance is determined by comparison with the results of molecular genetic, biochemical, and general physiological studies. Several studies have been conducted in this area. In the study by Breder et al. (2017), the MMSE demonstrated a sensitivity of only 45–65% for diagnosing Parkinson’s disease dementia (PDD). Faust-Socher et al. (2019) note that stable scores on the MoCA, MMSE and SCOPA-Cog in patients with PD without dementia cannot be taken as evidence of the absence of cognitive decline. Tests from the MMSE, MoCA and FAB categories are generally characterized by limitations relating to the social characteristics of the subjects, floor and ceiling effects, the guidance effect and learning (Atramentova, 2023). For instance, younger men from urban backgrounds with more years of education tend to perform better on the MMSE.

The observed simultaneous statistically significant improvements in motor (UPDRS II/III) and cognitive (MMSE, MoCA, SMR latency) functions following EGb 761 adjunctive therapy suggest a systemic effect. This clinical benefit aligns with the known multi-mechanistic profile of the extract, which includes modulation of neurotransmitter systems and promotion of neuroplasticity, both of which are critical for controlling movement and executive function. Specifically, the improved motor performance and reaction time (SMR) may be related to the reported effects of EGb 761 in animal models, where it increased dopamine levels (Smith et al., 1988).

### Motor symptoms

Four tests were conducted to assess the effects of EGb 761 on motor functions: UPDRS II (activities of daily living), UPDRS III (motor examination), motor tempo and SMR.

Our study indicates that EGb 761 treatment in combination with basic antiparkinsonian therapy was associated with faster analysis of sensory information and decision-making on one side of the body. It also improved the UPDRS results.

### Telomere lengths

Telomere shortening can serve as a marker of premature aging, age-related disorders, infectious and neurodegenerative diseases (Asghar et al., 2022; Schürks et al., 2014). In combination with mitochondrial dysfunction, cell cycle arrest and proinflammatory phenotype, telomere length are markers of cellular stress and senescence, and associated with age-related disorders and neurodegenerative diseases (Asghar et al., 2022). In our study, introducing EGb 761 into basic antiparkinsonian therapy was not associated with statistically significant changes in telomere length, although there was a trend towards elongation.

This finding may indicate that the intervention period was insufficient for measurable telomere remodeling, since telomere dynamics generally occur slowly and may require prolonged exposure. The observed tendency toward elongation may suggest a potential indirect effect mediated by reduced cellular stress and improved mitochondrial homeostasis.

### Number of mtDNA copies

The number of mtDNA copies increases with advancing age. This is believed to be a compensatory mechanism that maintains the amount of wild-type mtDNA, offsetting the functional effects of accumulated mtDNA mutations (Asghar et al., 2022). An increase in mtDNA copy number is a typical response to mitochondrial dysfunction, including complex I defects. This tendency is also observed in individuals carrying heterozygous PRKN variants, suggesting it may be a prerequisite for PD onset (Castelo Rueda et al., 2023). In general, it can be said that the number of mtDNA copies increases in the blood of patients with PD. This is traditionally interpreted as a consequence of impaired mitochondrial clearance or as a compensatory increase in mitochondrial biogenesis, which partially restores the insufficient supply of ATP to cells and maintains normal mitochondrial function (Ortega-Vázquez et al., 2023; Castelo Rueda et al., 2023). Studies have shown that, in SN neurons the number of mtDNA copies increases with age, thereby maintaining the mtDNA pool despite the accumulation of deletions. However, this regulatory mechanism is not always effective in individuals with PD, potentially leading to a reduction in total mtDNA levels (Asghar et al., 2022; Li et al., 2012). In our study, the addition of EGb 761 to the baseline antiparkinsonian therapy resulted in a statistically significant decrease in mtDNA copy number.

In contrast to reports suggesting an increased mtDNA copy number in patients with PD (often interpreted as a compensatory response to mitochondrial dysfunction), our study observed a statistically significant decrease in mtDNA copy number following EGb 761 administration. This finding is interpreted as evidence supporting the hypothesis that EGb 761 facilitates the removal of dysfunctional mitochondria through the activation of mitophagy, potentially normalizing the overall mitochondrial load in peripheral blood mononuclear cells (PBMCs). This interpretation aligns with data indicating that ginkgo components can influence mitochondrial clearance pathways (Wang et al., 2019).

### The state of the antioxidant system in patients with Parkinson’s disease

It has been established that oxidative stress is one of the factors responsible for the initiation and progression of PD. The main antioxidant enzymes present in the human body that protect against ROS cytotoxicity are superoxide dismutase (SOD), catalase (CAT) and glutathione peroxidase (GPx) (Abraham et al., 2005; Nikolova and Mancheva, 2013).

In rat studies, EGb 761 increased the enzymatic activity of SOD and GPx and decreased MDA levels (Md et al., 2022; Li et al., 2019). Similar results were obtained in mice (Feng et al., 2009).

Of all the enzymes in the antioxidant system, superoxide dismutase (SOD, EC 1.15.1.1) is considered to be part of the primary response to the development of oxidative stress (Miletić-Vukajlović et al., 2020; de la Torre et al., 1996). Several studies have demonstrated a reduction in SOD activity in patients with PD (Abraham et al., 2005; Miletić-Vukajlović et al., 2020; Nikolova and Mancheva, 2013; de la Torre et al., 1996; Tong et al., 2018). Information is available regarding the decrease in mitochondrial SOD2 activity associated with complete or partial Parkin deficiency (Castelo Rueda et al., 2023). A meta-analysis of 17 studies, six of which contained data on SOD activity, demonstrated that patients with PD had lower levels of SOD antioxidant activity than controls (Khan and Ali, 2018). This decrease is interpreted as a consequence of the prolonged inhibition of the body’s antioxidant system, resulting in the accumulation of mutations in genes and the oxidative modification of proteins (including enzymes), which decreases their activity and is comparable to that observed in chronic diseases (Miletić-Vukajlović et al., 2020). In our study, introducing EGb 761 to baseline antiparkinsonian therapy did not affect SOD activity.

CAT (EC 1.11.1.6) activity was both reduced in patients with PD (Abraham et al., 2005; de la Torre et al., 1996) and increased compared to healthy individuals (Miletić-Vukajlović et al., 2020; Nikolova and Mancheva, 2013). A meta-analysis including 17 studies, five of which contained data on CAT activity, showed that patients with PD had lower levels of CAT antioxidant activity than controls (Khan and Ali, 2018). Like SOD activity, CAT activity did not change after a month of adjunctive antiparkinsonian therapy.

The formation of advanced lipoxidation end products (ALEs) and advanced glycation end products (AGEs) is caused by oxidative stress (Sharma et al., 2020; Li et al., 2012). However, AGE formation can also cause oxidative stress, creating a positive feedback loop that increases ROS generation (Li et al., 2012; Perrone et al., 2020; Kang et al., 2022). AGEs produce ROS, which contribute to redox imbalance and can ultimately trigger AGE formation (Kang et al., 2022). This loop is primarily explained by the activation of the inflammation cascade through the RAGE signaling pathway in microglia and other cells carrying this receptor, such as neurons and astrocytes (Zgutka et al., 2023; D’Cunha et al., 2022; Yamagishi and Matsui, 2018; Chrysanthou et al., 2022; Anwar et al., 2021; Kuzan, 2021). Those changes in cellular signaling result in NF-κB-dependent synthesis of pro-inflammatory and inflammatory mediators such as cytokines, growth factors, adhesion proteins and acute phase proteins (Zgutka et al., 2023; Perrone et al., 2020). Post-translational modifications of AGE proteins affect the biomechanical and functional properties of macromolecules, including their structure, enzymatic activity and biological half-life. Thus, it alters the normal course of numerous physiological processes at levels ranging from the molecular to the organismic (Almengló et al., 2022). The electrical charge of the protein changes, which distorts its ability to interact normally with low-molecular-weight compounds and other macromolecules (Zgutka et al., 2023). AGEs have been shown to cause *α*-synuclein aggregation in an *in vitro* system (Li et al., 2012). In general, AGE formation reactions alter the structure and function of proteins, removing them from normal physiological processes (Kang et al., 2022). We did not observe any statistically significant changes in the relative concentration of AGEs following 1 month of EGb 761 antiparkinsonian therapy.

Glutathione (GSH) is a tripeptide consisting of the amino acids *γ*-glutamylcysteinylglycine. The reduced form of GSH contains an SH group that plays a key role in the cellular antioxidant defense system. It is the most abundant antioxidant molecule in the central nervous system (CNS) (Asanuma and Miyazaki, 2021). In patients with PD, GSH levels in the *substantia nigra* (SN) are reduced to approximately 40% of the control values (Asanuma and Miyazaki, 2021; Sanchez et al., 2021). Generally, a decrease in GSH is considered an early indicator of PD progression (Wang et al., 2021). EGb 761 administration alongside basic antiparkinsonian therapy did not increase the concentration of one of the key low-molecular-weight antioxidants; in fact, we observed a statistically significant decrease.

MDA is the end product of membrane lipid peroxidation. During the development of oxidative stress, polyunsaturated fatty acids from membrane phospholipids are the main target substrates for ROS (Nikolova and Mancheva, 2013). Experimental reports and meta-analyses have shown that MDA levels are significantly elevated in patients with PD (Wei et al., 2018; Khan and Ali, 2018; Nikolova and Mancheva, 2013). Our study showed that EGb 761 treatment in combination with basic antiparkinsonian therapy did not lead to a decrease in oxidative stress markers. In contrast, we observed a statistically significant increase in MDA levels in patients with PD.

The observed molecular profile, specifically the statistically significant increase in the lipid peroxidation marker MDA and the decrease in the key low-molecular-weight antioxidant GSH/GSSG, appears counterintuitive for an agent known for its antioxidant properties, particularly when concurrent with clinical improvement. However, this signature may reflect the dynamic initiation of cellular clearance rather than a failure of the antioxidant defense. Given that the MDA increase and GSH/GSSG decrease occurred alongside the reduction in mtDNA copies, these transient changes might represent temporary metabolic stress induced by the rapid removal of dysfunctional mitochondria via activated mitophagy, a process known to involve the transient signaling of reactive oxygen species (ROS) before cellular repair is completed (Shefa et al., 2019).

### Energy metabolism

Cellular aging is manifested by changing in the metabolic profile. This involves a switch from oxidative phosphorylation to anaerobic glycolysis, resulting in a progressive decrease in the synthesis of macroenergic molecules. 30–36 ATP molecules can be generated from one glucose molecule through oxidative metabolism, whereas only two ATP molecules are formed during anaerobic glycolysis (Feng et al., 2016). However, the relatively low bioenergy efficiency of glycolysis is offset by the intensity of metabolic flux, which is sufficient even for rapid cell proliferation (Ishida et al., 2020).

PD is characterized by impaired energy metabolism and a decrease in ATP levels (Li J. et al., 2022). Restoring ATP levels in dopaminergic neurons requires a metabolic switch to aerobic glycolysis and increased LDH (EC 1.1.1.27) activity to form NADPH (Li J. et al., 2022; Akter et al., 2023; Ravera et al., 2019). This may be due to increased HIF-1*α* expression, which has been reported to be elevated in the *substantia nigra pars compacta* (SNpc) of patients with sporadic PD compared to control subjects (Li J. et al., 2022). Experimental overexpression of α-synuclein has been shown to significantly increase ROS and LDH levels (Wang et al., 2010). Empirical evidence suggests that lactate levels are abnormally high in the cerebrospinal fluid of patients with late-onset PD (Yang et al., 2024). However, our study did not reveal any statistically significant changes in LDH activity in peripheral blood mononuclear cells (PBMCs) or plasma in patients with PD after EGb 761 treatment alongside basic antiparkinsonian therapy.

Although the literature suggests impaired energy metabolism in PD, our study did not reveal any statistically significant changes in LDH activity in peripheral blood mononuclear cells (PBMCs) or plasma. This lack of change in a peripheral marker over a short period may indicate that the metabolic effects of EGb 761 are confined to central nervous system tissue, or that peripheral LDH activity is not a sensitive immediate indicator of the drug’s short-term influence on energy pathways in PD.

### Mechanism of action of EGb 761

*Ginkgo biloba* has an exceptionally wide range of biologically active substances in its chemical composition, including ginkgetin, bilobetin, 5-methoxybilobetin, sciadopitysin, amentoflavone, kaempferol, quercetin, isorhamnetin, rutin, myricetin, luteolin, apigenin, ginkgolides A, B, C, J, M, N and P, and bilobalide (Md et al., 2022; Nowak et al., 2021; Christen, 2004; Mahadevan and Park, 2008; Feng et al., 2009). It is well documented that other compounds within the extract, such as proanthocyanidins, are potent radical scavengers (Kulić et al., 2022). Consequently, the therapeutic action of EGb 761 is widely considered to be multi-mechanistic, potentially involving neuroplasticity, anti-inflammatory properties, and effects on neurotransmitter systems (such as dopamine) in addition to its known antioxidative properties (Shah et al., 2003; Fehske et al., 2009; Gargouri et al., 2018; Tchantchou et al., 2007).

A complex of laboratory methods for studying molecular, genetic and biochemical markers revealed statistically significant changes in the relative number of mtDNA copies (decrease), GSH/GSSG concentration (decrease) and MDA (increase). It is known that ginkgolic acids are capable of inducing mitophagy (Wang et al., 2019). However, the standardized extract EGb 761 only contains negligible amounts of these compounds (Kulić et al., 2022).

It is possible that the selected dose or duration of treatment was insufficient to produce a statistically significant attenuation of oxidative stress, and that the observed reduction in mtDNA copy number reflects the natural loss of organelles rather than activated mitophagy, or mitophagy is indeed activated, but due to other components present in the preparation.

As the main source of ROS, mitochondria have a negative feedback mechanism aimed at eliminating them when intracellular ROS concentrations increase (Frank et al., 2012; Liu et al., 2022). Mitophagy is one such mechanism of this negative feedback. This selective form of autophagy eliminates dysfunctional mitochondria and their damaged components, resulting from their involvement in oxidative stress reactions (Wang et al., 2019; Lin Y.-C. et al., 2022). Therefore, as oxidative stress progressed, EGb 761 treatment may have activated or significantly enhanced the elimination mechanisms of ROS sources.

Regarding the systemic improvements detected by clinical scales, it cannot be excluded that they may also result from the drug’s action on the dopaminergic system (Shah et al., 2003; Yoshitake et al., 2010). Specifically, in animal models (rats), chronic but not acute treatment with EGb 761 increased extracellular dopamine and noradrenaline—but not serotonin—in the prefrontal cortex, with only a marginal effect in the striatum. This effect is mainly linked to the flavonol glycoside fraction and, to a lesser extent, to ginkgolides, whereas bilobalide appears to have no effect (Yoshitake et al., 2010). Simultaneously, EGb 761 acts as a neuroprotective factor, preventing stress-induced elevations of dopamine, norepinephrine, serotonin, and corticosterone, restoring them to normal levels (Shah et al., 2003). The molecular mechanisms involve the inhibition of norepinephrine (NET), serotonin (SERT), and dopamine (DAT) transporters and MAO activity *in vitro*, although relatively high concentrations are required to inhibit MAO-A and MAO-B. However, after chronic treatment, only norepinephrine uptake was significantly reduced *in vivo*, with no effect on MAO activity (Fehske et al., 2009). Additionally, EGb 761 reduces the number of peripheral benzodiazepine receptors in the adrenal glands, thereby decreasing corticosterone secretion. Primarily, the observed effects were associated with improved cognitive function (Shah et al., 2003).

In this context, the therapeutic potential of EGb 761 does not appear to be limited to its antioxidant properties and monoamine modulation. As data from the literature indicate, the extract also possesses pronounced anti-inflammatory activity: in cell cultures, EGb 761 has been shown to reduce neuroinflammatory activation by targeting the COX/PGE2 pathway (Gargouri et al., 2018) and to decrease the release of TNF-*α*, IL-6, CXCL2, CXCL10, CCL2, and CCL3 (Sun et al., 2024).

Current concepts of the pathogenesis of PD point to a significant role of neuroinflammatory processes, which not only accompany but may also accelerate the death of dopaminergic neurons. Neuroinflammation in this context is frequently associated with cellular senescence of glial and neuronal cell types (Martin-Ruiz et al., 2019). Systemic inflammation also contributes to this, arising and intensifying with age, as evidenced by higher levels of circulating pro-inflammatory cytokines (i.e., IL-1β, IL-6, and TNF-α) (Gonzales et al., 2022). Neuroinflammatory mechanisms mediated by activated glial and peripheral immune cells induce oxidative stress and apoptosis via cytokine receptors, which over time leads to neuronal death and progression of PD (Schürks et al., 2014). This provides the basis for establishing a link between inflammatory processes and rapid deterioration of motor functions (Martin-Ruiz et al., 2019), the reduction of which probably contributed to the improvement of motor indicators in our patients.

Moreover, these findings suggest not only a certain reduction in the intensity of pathogenetic processes but also the potential activation of regenerative mechanisms. In mouse models of Alzheimer’s disease, EGb 761 was shown to enhance hippocampal neurogenesis (Tchantchou et al., 2007), while bilobalide and quercetin, components of the extract, significantly enhanced hippocampal neuronal proliferation and synaptogenesis and protected against Aβ oligomer-induced synaptic loss (Tchantchou et al., 2009), effects that are likely mediated by CREB activation (Tchantchou et al., 2007). Based on these findings and supporting literature, EGb 761 is considered a promising agent capable not only of influencing various links of pathogenesis but also, potentially, of initiating sanogenetic processes.

This study has several limitations. First, the absence of a control group: the before/after design on a single group of patients cannot rule out the influence of concomitant therapy (in particular, L-DOPA), the natural course of the disease, the psychological effect of additional medical observation, or the placebo effect. Second, the sample size was small (only 17 patients) which significantly limited the statistical power and generalizability of the results. Third, the treatment period was short, lasting for 1 month. Finally, the lack of clear criteria for determining the baseline observation point may have affected the interpretation of patient progression and reduced the reproducibility of the study design.

## Conclusion

The results of this study support the consideration of EGb 761 as a potentially beneficial adjunctive component of complex symptomatic therapy for Parkinson’s disease. The present study observed improvements in clinical and neurophysiological tests, including the UPDRS II, UPDRS III, MMSE, MoCA, and SMR, following a month-long treatment with EGb 761 alongside basic anti-Parkinsonian therapy. The results also corresponded to improvements in patients’ cognitive function and quality of daily living. Furthermore, the observed effects may be associated with the ability of EGb 761 to attenuate oxidative stress and mitigate pathological processes via the activation of mitophagic mechanisms. This assumption is supported by the tendency toward telomere lengthening, a decrease in mitochondrial DNA copy number, and concurrent improvement in cognitive test performance.

## Data Availability

The original contributions presented in the study are included in the article/supplementary material, further inquiries can be directed to the corresponding author.
